# CD8^+^ T Cell Fate and Function Influenced by Antigen-Specific Virus-Like Nanoparticles Co-Expressing Membrane Tethered IL-2

**DOI:** 10.1371/journal.pone.0126034

**Published:** 2015-05-06

**Authors:** Daniela Wojta-Stremayr, Alina Neunkirchner, Bharani Srinivasan, Doris Trapin, Klaus G. Schmetterer, Winfried F. Pickl

**Affiliations:** 1 Institute of Immunology, Center for Pathophysiology, Infectiology and Immunology, Medical University of Vienna, Vienna, Austria; 2 Christian Doppler Laboratory for Immunomodulation, Vienna, Austria; Indiana University, UNITED STATES

## Abstract

A variety of adjuvants fostering humoral immunity are known as of today. However, there is a lack of adjuvants or adjuvant strategies, which directly target T cellular effector functions and memory. We here determined whether systemically toxic cytokines such as IL-2 can be restricted to the site of antigen presentation and used as ‘natural adjuvants’. Therefore, we devised antigen-presenting virus-like nanoparticles (VNP) co-expressing IL-2 attached to different membrane-anchors and assessed their potency to modulate CD8^+^ T cell responses *in vitro* and *in vivo*. Efficient targeting of IL-2 to lipid rafts and ultimately VNP was achieved by fusing IL-2 at its C-terminus to a minimal glycosylphosphatidylinositol (GPI)-anchor acceptor sequence. To identify optimal membrane-anchor dimensions we inserted one (1Ig), two (2Ig) or four (4Ig) immunoglobulin(Ig)-like domains of CD16b between IL-2 and the minimal GPI-anchor acceptor sequence of CD16b (GPI). We found that the 2IgGPI version was superior to all other evaluated IL-2 variants (IL-2v) in terms of its i) degree of targeting to lipid rafts and to the VNP surface, ii) biological activity, iii) co-stimulation of cognate T cells in the absence of bystander activation and iv) potency to induce differentiation and acquisition of CD8^+^ T cell effector functions *in vitro* and *in vivo*. In contrast, the GPI version rather favored memory precursor cell formation. These results exemplify novel beneficial features of membrane-bound IL-2, which in addition to its mere T cell stimulatory capacity include the induction of differential effector and memory functions in CD8^+^ T lymphocytes.

## Introduction

All clinically successful vaccines available as of today confer protection by inducing long lasting, protective, affinity-matured antibodies [1]. Although T cells are presumed to be critically involved in humoral vaccine responses, efforts to engage T cell responses by vaccines have for the most part been met with mixed success [2]. In some measure this may be attributable to the greater intrinsic variability of the T cell response due to MHC polymorphism. In certain settings, for example in the development of vaccines for intracellular pathogens or tumor cells, T cell engagement has been thought to be highly desirable if not essential [3, 4]. However, our knowledge regarding adjuvants or adjuvant strategies, which directly target T cellular effector functions and memory is still scarce. This is particularly true for immune responses based on CD8^+^ cytotoxic T cells. In a related aspect, it would be highly interesting to better understand how CD8^+^ T cell efficacy is determined by i) release of cytotoxic compounds, ii) production of IFN-γ, and iii) differential expression of homing (CD62L) and memory markers (CD44, Ly6c, CD127).

One way to modulate CD8^+^ T cell-based immunity would be to co-administer CD8^+^ T cell-tropic differentiation and growth factors along with antigens of interest. Besides fostering humoral immunity, such factors also have been shown to co-stimulate strong cellular immune responses, which is a *conditio sine qua non* in targeting facultative intracellular pathogens or tumor cells. In that respect, cytokines such as IL-2, IL-12 and GM-CSF can be regarded as ‘natural adjuvants’. In fact, cytokines have been applied in the past to improve immunity [5, 6], however, their application in soluble form often lead to undesired systemic adverse reactions [7, 8]. This is mainly due to ‘overdosing’ of cytokines, which is necessary in order to reach pharmacologically relevant concentrations of the soluble agents in target tissues.

To restrict the function of cytokines to the site of antigen-presentation but nevertheless guarantee high immunomodulatory cytokine concentrations locally, we have developed a simple and versatile system for the decoration of virus-like nanoparticles (VNP) with biologically active cytokines in the past [9–12]. The particulate nature of the platform allows for a concerted action of antigen(s) and the immunomodulatory compound. Moreover, it ensures high local concentration of the cytokine, allowing to reduce to overall amount of cytokine applied. In addition, the particulate nature of the VNP platform might retard the systemic diffusion of the attached cytokine. One of the key cytokines for T cell development and function, i.e. IL-2, supports cell cycle progression, proliferation and survival of T cells and has a strong impact on both early and terminal-effector differentiation of CD8^+^ T cells [13–16]. IL-2 is primarily produced by activated CD4^+^ and to a lesser extent by CD8^+^ T cells [17]. Whether CD4^+^ T cell derived IL-2 is essential for CD8^+^ memory formation is currently debated. In fact, *Schoenberger* and colleagues have shown that CD4^+^ T cells rather provide CD40/CD40L-based help to cognate APC, which in turn stimulate CD8^+^ T cells via CD70/CD27 interaction to produce autocrine IL-2 and develop into memory cells [18, 19].

To better understand and to find novel ways to actively and directly influence CD8^+^ T cell differentiation we here generated and biochemically and functionally evaluated membrane-anchored IL-2 variants co-expressed with antigen-specific pMHC on the same VNP-based plasma membrane matrix. Specifically, we analyzed the co-stimulatory potency of differentially anchored forms of IL-2 to induce antigen-specific CD8^+^ T cell activation and expansion. Furthermore, we studied the influence of IL-2 variants on terminal effector and memory precursor-like cell formation and function. Finally, we evaluated the potential of IL-2 decorated antigen-specific (as)VNP to induce cytotoxic effector functions *in vivo* and studied the memory phenotype of adoptively transferred T cells pre-stimulated with IL-2v decorated VNP.

## Materials and Methods

### Ethics statement

Animals were kept under conventional conditions and used for experiments according to the animal ethics guidelines of the Medical University of Vienna. The project was approved by the ethics committee of the Medical University of Vienna.

### Mice

Animals, C57BL/6-J (CD45.2^+^, Harlan SRL, San Pietro al Natisone, Italy), congenic C57BL/6-J (CD45.1^+^; European Mouse Mutant Archive, Munich, Germany) and P14 TCR transgenic mice specific for lymphocytic choriomeningitis virus (LCMV) glycoprotein 33–41 (LCMV-GP_33-41_) in context with H-2D^b^ (sub-line 327, ETH Zurich, Zurich, Switzerland; [20]) were used for experiments at 8–16 weeks of age.

### Cell lines and primary cells

HEK-293 (human embryonic kidney, American Type Culture Collection (ATCC), Manassas, VA), EL-4 wt (ATCC) and EL-4 LCMV-GP (ATCC) cells were cultured in IMDM (PAA Laboratories GmbH, Pasching, Austria) plus 10% fetal calf serum (FCS) (Invitrogen, Carlsbad, CA) supplemented with 2 mM L-glutamine (PAA), 50 μM 2-mercaptoethanol and 15 μg/ml gentamicin sulfate (PAA). EL-4 LCMV-GP cells were generated by transfecting EL-4 cells with an LCMV-GP expression plasmid (kindly provided by Dr von Laer, Hamburg Germany) using a modified calcium-phosphate precipitation method as described [9]. HT-2 cells (ATCC) and primary P14 TCR transgenic splenocytes were maintained in RPMI (PAA) plus 10% FCS supplemented with 2 mM L-glutamine, 100 μM 2-mercaptoethanol, 50 μg/ml gentamycin sulfate, 1 mM sodium pyruvate, non-essential amino acids, 10 mM HEPES buffer pH 7.3 (Invitrogen). HT-2 culture medium was supplemented with human IL-2 (150 U/ml) (PeproTech, London, UK).

### Plasmid construction

Murine IL-2 was amplified from pcDmouseIL-2 [MT-1] (ATCC 39892; American Type Culture Collection, Manassas, VA), using the primers IL-2 for and IL-2 rev (see S1 Table) and inserted into pEAK12 (Edge Biosystems, Gaithersburg, MD) containing a minimal GPI-anchor acceptor sequence [10]. The primer pairs 1IgGPI for and CD16b rev and 2IgGPI for and CD16b rev were used to amplify the membrane proximal Ig domain or both Ig domains of human CD16b [21] designated 1IgGPI or 2IgGPI, respectively, flanked by a *Nhe*I and a enhanced stop codon (TAAA) followed by a *Not*I restriction site at its 5’- or 3’-ends, respectively. Sequences encoding novel anchors were ligated into pEAK12_IL-2::GPI, replacing the minimal GPI-anchor acceptor sequence [9]. The 4IgGPI-anchor was generated by duplicating 2IgGPI CD16b with primers 2IgGPI for and 4IgGPI rev. Insertion of a *Spe*I restriction site into the 5’ terminus of the CD16b cDNA allowed insertion of the fragment into *Nhe*I digested IL-2::2IgGPI. Constructs were designated IL-2::1IgGPI, IL-2::2IgGPI and IL-2::4IgGPI (see S1 Fig). Loss of function variants of IL-2 were generated as described for human IL-2 [22] and designated IL-2(A)::GPI and IL-2(A)::2IgGPI. Abrogation of Fcγ binding activity [23] was achieved by site directed mutagenesis of codon 112 of hCD16b using the primers 16bmut for and 16bmut rev (Phusion site directed mutagenesis kit, Fisher Scienitic GmbH, Vienna, Austria). Constructs were designated IL-2::2Ig(F)GPI and IL-2(A)::2Ig(F)GPI and their integrity confirmed by DNA-sequencing. Moloney murine leukemia virus (MoMLV) *gag*-*pol* (OGP) was expressed using pMD.gagpol [24]. The pEAK12 expression constructs coding for mouse H-2D^b^::GPI, β2m, and the peptide minigene coding for LCMV-GP_33-41_ have been described previously [9].

### Flow cytometric analyses

Analyses of cell surface expressed molecules were performed as described [25] using the mAbs listed in S2 Table, and propidium iodide to exclude dead cells. For analysis of stained cell samples, either a FACSCalibur equipped with the CellQuest software or a LSR Fortessa equipped with the Diva software were used (Becton Dickinson). Data are displayed as overlay histograms or two-parameter dot-plots with the channel numbers indicated in each plot. IgG binding capacity of CD16b was determined by incubating cells with 10 μg/ml Beriglobin P (CSL Behring, Vienna, Austria) followed by staining with PE-conjugated anti-human Ig antibodies (Jackson ImmunoResearch Europe Ltd, Suffolk, UK). Intracellular IFN-γ-levels were determined after treatment of cells with PMA (0.05 μg/ml) plus ionomycin (0.1 μg/ml) (Sigma-Aldrich, Vienna, Austria) and Golgi-Stop (1:1500, Becton Dickinson, Palo Alto, California) for 6 hours and using Fix and Perm (An der Grub, Kaumberg, Austria). CD8^+^ T cell numbers in *in vitro* cultures were determined by comparing viable cell numbers with CountBright Absolute Counting Beads (Life Technologies, Molecular Probes, Eugene, Orlando).

### Generation of virus-like nanoparticles (VNP)

For the generation of VNP, HEK-293 cells were transiently transfected using a modified calcium-phosphate precipitation method [26] as described [9]. Briefly, one day prior to transfection, HEK-293 cells were seeded onto 100 mm culture dishes at a concentration of 1.2x10^6^ cells and were transfected the following day with 2 ml transfection mix. DNA mixes (30 μg per dish) for transfection included 5–10 μg of the MoMLV original *gag-pol* (OGP) encoding plasmid pMD.gagpol and 5 μg of the expression plasmids in pEAK12 encoding for the proteins as indicated. If necessary, DNA amounts were adjusted with control vector (empty pEAK12 vector or where indicated full length CD16b in pEAK12) to yield a total of 30 μg. One day after transfection, the medium was changed. Supernatants were harvested three days after transfection, cleared of cellular debris by filtration (0.45 μm filters, Sarstedt, Nümbrecht, Germany) and concentrated by ultracentrifugation (Optima LE-80K, Beckman Instruments, Palo Alto, CA), using a SW41 Ti rotor, at 1x10^5^ g at 4°C for 1 h followed by two washing steps in PBS. Alternatively, VNP were concentrated by Centricon Plus-70 (Millipore GesmbH, Vienna, Austria), followed by two PBS washes by ultracentrifugation. VNP concentrations were determined by bicinchoninic acid assay (Micro BCA, Pierce, Thermo Fisher Scientific, Rockford, IL) and adjusted as indicated in individual experiments. VNP were used directly or stored at—80°C before use, which did not alter their function [25]. One microgram of VNP contained less than 0.03 EU of endotoxin as determined by limulus amebocyte lysate assay (LAL) (Lonza Group Ltd., Basel, Switzerland).

### Biochemical analyses of producer cells and VNP

HEK-293 cell lysates were prepared by incubating 1x10^6^ cells in 10 μl lysis buffer containing 1% NP-40, 1 mM EDTA, 1 mM PMSF, 20 μg/ml aprotinin and 20 μg/ml leupeptin in MBS (25 mM MES, 150 mM NaCl) for one hour on ice. Subsequently, lysates were centrifuged at 80 g to remove insoluble material at 4°C for 10 min and mixed with 4 x Laemmli sample buffer. VNP were concentrated, washed and the protein concentration determined as described above, followed by re-suspension in 1 x Laemmli sample buffer. Cellular (0.6 μg/lane) and VNP (1.5 μg) lysates along with a defined amount of mIL-2 (33 ng (165 U)/lane) were resolved by 4–20% SDS-PAGE (Anamed, Darmstadt, Germany). Subsequently, proteins were transferred to nitrocellulose membranes (Biorad, Hercules, CA) and subjected to immunoblotting (antibodies listed in S2 Table). Blots were developed with a luminol based indicator system (Western Lightning, Perkin Elmer, Boston, MA) and exposed to x-ray films (Eastman Kodak, Rochester, NY).

### Isopycnic separation of producer cell lysates

Lipid rafts were prepared and resolved by SDS-PAGE as described previously [9, 27]. Briefly, 293T cells were transfected with the indicated expression plasmids and the retroviral gag-pol vector. After 48 hours cells were lysed in a 1% Triton X-100 containing buffer. Subsequently, lysates were Dounce-homogenized, cleared from particulate material by centrifugation at 4°C and 100 x g for 10 min, mixed with an equal volume of 80% (wt/vol in MES-buffered saline) sucrose and placed in a SW55 centrifuge tube (Beckmann). Samples were then overlaid with 2 ml of 30% sucrose followed by 1 ml of 5% sucrose and spun at 200,000 g and 4°C for 16 to 18 hours. The gradients were collected in 0.5 ml steps from the top to the bottom. Top fractions 2–3 typically contain the detergent-insoluble proteins, which become visible at the 5/30% interface, while soluble cellular proteins become concentrated in bottom fractions 6–9 of the gradients. Proteins were blotted onto nitrocellulose membranes (Biorad), subjected to immunoblotting (antibodies listed in S2 Table) and developed as described above.

### Dot blot analyses

Supernatants were concentrated by ultracentrifugation (SW41 Ti rotor, 1x10^5^ g, 1 h), washed once in PBS and pellets re-suspended in 100 μl PBS. Three fold dilutions of VNP suspensions were applied onto nitrocellulose membranes (Biorad) under mild vacuum (immunodot apparatus, V&P Scientific, San Diego, CA). After fixation in blotting buffer the membranes were incubated with *gag*-specifc mAb R187. Blots were developed as described above. Immuno-reactive proteins were detected on a Vilber Lourmat apparatus (Torcy, France) with the aid of the ChemiCapture5000 software. Densitometric quantification was performed using the Bio1D Software (Vilber Lourmat).

### HT-2 proliferation bioassays

HT-2 cells (5x10^3^) were incubated in 96-well flat-bottom tissue culture plates for 48 hours with titrated amounts of VNP (starting at 50 μg/ml), decorated with either IL-2v or control molecules as indicated. Cells were pulsed with methyl-[^3^H]-thymidine (1 μCi per well) for another 16 hours and processed as previously described [9]. Medium alone served as control. ED_50_-values were determined with the aid of the GraphPad Prism program (GraphPad Software, Inc. La Jolla, CA).

### P14 TCR transgenic T cell stimulation assays

Splenocytes (2x10^5^) of P14 TCR transgenic mice, subline 327, in which 80–90% of CD8^+^ T cells expressed TCR Vβ2/Vα8, were incubated in 96-well flat-bottom plates with VNP expressing H-2D^b^::GPI along with murine β2 microglobulin in the presence (antigen-specific, (as)VNP) or absence (antigen non-specific, (ans)VNP) of a peptide minigene coding for the immunodominant CD8^+^ T cell epitope LCMV-GP_33-41_ [9] and where indicated also functional (IL-2) or non-functional (IL-2(A)) IL-2 variants (IL-2v) on the same particle for three days and proliferation was assessed [9]. VNP did not express classical co-stimulatory or adhesion molecules. When indicated the cells were additionally supplemented with various amounts of soluble, recombinant IL-2 (PeproTech). Medium alone served as control. Alternatively, purified CD8^+^ T cells (CD8a^+^ T cell Isolation Kit II, Miltenyi, Bergisch Gladbach, Germany) were used in individual experiments. After 48, 72 and 96 h, cell culture supernatants were harvested and the concentration of IFN-γ was determined as described [28]. The amount of IFN-γ production on a cellular level was determined 96 hours after stimulation as depicted in immunofluorescence analyses.

For re-stimulation experiments purified CD8^+^ T cells were stimulated in the primary stimulation with VNP decorated as indicated for 7 days. Cells were washed twice and cultured (5x10^5^ cells/ml) in the presence or absence of recombinant IL-7 (125 U/ml) (PeproTech) for additional 4 days. Subsequently, 1x10^4^ viable cells/200 μl were re-stimulated with (1x10^4^) irradiated (60 Gy) splenocytes, which had been pulsed with 0.1–100 ng/ml LCMV-GP_33-41_ peptide (ProImmune, Cambridge, UK) overnight. After 24 hours supernatants were harvested and cytokine concentrations determined by multiplex analysis (Luminex 100IS; Biomedica, Vienna, Austria). Subsequently, proliferation was assessed by methyl-[^3^H]-thymidine up-take [9].

### Determination of antigen-specific CD8^+^ T cell cytotoxicity

CFSE labeled (Molecular Probes) P14 splenocytes (2x10^5^) were incubated in 200 μl with VNP (8 μg) decorated with H-2D^b^::GPI, μ2m, LCMV-GP_33-41_ peptide minigene and either IL-2::GPI, IL-2(A)::GPI, IL-2::2Ig(F)GPI or IL-2(A)::2Ig(F)GPI. After 48 hours, cells were harvested and co-cultured in titrated amounts with a constant number of irradiated (60 Gy) EL-4 LCMV-GP target cells that had been labeled with cell proliferation dye (CPD) eFluor670 (0.1 μM CPD^low^, eBioscience, Vienna, Austria). Wildtype EL-4 cells were labeled with 1 μM CPD (CPD^high^) and were used as control target cells. After 20 hours specific target cell lysis was determined by comparing the ratio of CPD^high^ and CPD^low^ cells. Percent specific lysis relative to LCMV-GP_33-41_ stimulated cells was calculated as follows [effector cell induced lysis (% reduction of EL-4 GP cells compared to EL-4 wt cells)—spontaneous lysis (%)/maximum lysis (% reduction of peptide stimulated effector cells)—spontaneous lysis (%)] x 100. Maximum lysis was determined by LCMV-GP_33-41_ peptide activated splenocytes. Alternatively, the cytolytic activity was determined by standard ^51^Cr-release assays. Briefly, P14 splenocytes (2x10^5^) were incubated with 6 μg antigen-specific VNP decorated with IL-2v. EL-4 stably expressing LCMV-GP or wildtype EL-4 cells were labeled with 100 μCi of Na^51^CrO_4_ (PerkinElmer, Boston, MA) in 100 μl of medium at 37°C for 1 hour. After four washes 1x10^4^ EL-4 cells were co-cultured in triplicates in a 96-well plate with pre-stimulated splenocytes at effector to target cell ratios of 20, 10, 5, 2.5 at 37°C. After 6 hours the supernatants were collected and the radioactivity was determined in a γ-counter (Packard). The percentage specific lysis was determined as follows: [effector cell induced lysis (cpm)—spontaneous lysis (cpm)/maximum lysis (cpm)—spontaneous lysis (cpm)] x 100, and related to the specific lysis obtained with peptide-induced splenocytes.

### *In vivo* cytotoxicity assay

Purified P14 CD8^+^ (CD45.2^+^) T cells (2x10^5^ cells/mouse) were transferred intravenously (tail vein) into C57BL/6 (CD45.2^+^) recipient mice. Subsequently mice were either left untreated (-VNP) or immunized intravenously with 200 μg of antigen-specific VNP (+VNP) decorated with IL-2 variants as indicated. After 48 hours 2x10^6^ CPD labeled splenocytes obtained from congenic CD45.1^+^ donor mice pulsed with (5 μg/ml) LCMV-GP_33-41_ (CPD^dim^) overnight were transferred as target cells into the differentially treated recipient mice. CPD^bright^ labeled, mock peptide (FLU M1_58-66_) pulsed splenocytes served as control target cells. 48 hours later mice were sacrificed and spleens were analyzed for specific target cell lysis by means of flow cytometry. Specific lysis was calculated according to the formula

$$100-\left[\left({\text{CPD}}^{\dim/+\text{VNP}}/{\text{CPD}}^{\text{bright}/+\text{VNP}}\right)\;*100\right]/\left[\left({\text{CPD}}^{\text{dim}/-\text{VNP}}/{\text{CPD}}^{\text{bright}/-\text{VNP}}\right)*100\right]*100.$$

### Determination of memory phenotype of adoptively transferred CD8^+^ T cells pre-stimulated *in vitro* with IL-2v asVNP

Purified P14 CD8^+^ (CD45.2^+^) T cells were stimulated as described above. After six days of *in vitro* culture 1x10^6^ viable cells (determined by PJ staining) were intravenously (tail vein) transferred into C57BL/6 (CD45.1^+^) recipient mice. After 7 days of *in vivo* resting, mice were sacrificed and spleens and inguinal lymph nodes were isolated and subjected to flow cytometry to determine the amounts and memory phenotype of recovered CD8^+^ T lymphocytes.

### Statistical analyses

Statistical analyses were performed using GraphPad Prism 6 software (GraphPad Software, La Jolla, CA). Comparisons were performed by two-tailed t-test or Mann-Whitney U-test as indicated in the figure legends. For multiple comparisons normally distributed data were analyzed by one-way ANOVA and Tukey’s multiple comparison test or t-test followed by post hoc Bonferroni correction. In all other cases, the Kruskal-Wallis test and Mann-Whitney U-test followed by post hoc Bonferroni correction or Wilcoxon rank sum test were used. Statistical significant values are denoted as follows: *, p<0.05; **, p<0.01; ***, p<0.001.

## Results

### Membrane-anchor characteristics influence IL-2 expression levels and VNP formation

**Fig 1:**
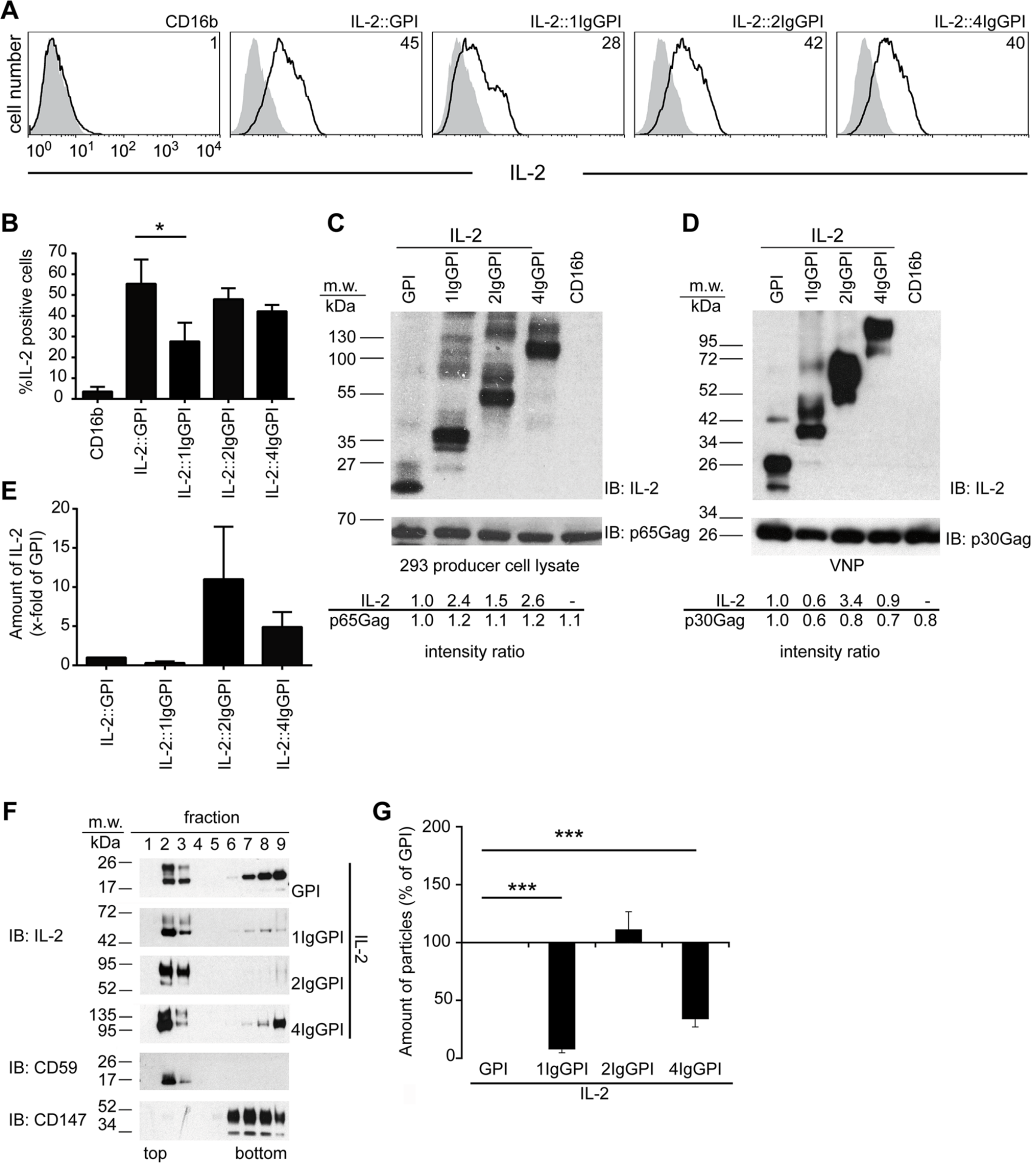
Expression and targeting of IL-2 fused to different membrane anchors of CD16b. (**A**) Surface expression levels of IL-2 fused to different membrane anchors on HEK-293 cells. HEK-293 producer cells were transfected with indicated plasmids and stained with IL-2-specific (black line) or non-binding control mAb (grey histograms), followed by flow cytometric analysis. Numbers indicate percent positive cells. (**B**) Percentage of IL-2 positive HEK-293 cells upon transfection with the indicated constructs. (**C** and **D**) Expression of IL-2v in HEK-293 producer cells and corresponding VNP preparations as detected with IL-2-specific antiserum. Anti-Gag was used as loading control. Bottom panels show quantification of band intensities (Image J 1.48 software) normalized to values obtained from whole cell lysates of IL-2::GPI transfectants and corresponding VNP. (**E**) Relative amounts of IL-2 targeted to VNP compared to IL-2::GPI VNP. (**F**) GPI-anchor attachment targets IL-2 into lipid rafts of HEK-293 cells. Triton X-100 lysates of HEK-293 cells were resolved by 5 to 40% sucrose gradients into nine fractions (top to bottom), subjected to SDS-PAGE, blotted and probed (IB) with IL-2-specific antiserum, CD59 (lipid raft targeting control) and CD147 (non-lipid raft targeting control) mAb. (**G**) Dot-blot analyses of relative amounts of particles secreted by HEK-293 producer cells transfected with indicated constructs using p30Gag immunoblotting (IB). Data are representative (A, C, D, F) or show the summary (B, E, G) of three (except six for IL-2::GPI and IL-2::1IgGPI) (A to D), four (E, G) and two (F) independent experiments. * p < 0.05, ***, p < 0.001; Kruskal-Wallis test and Mann-Whitney-U-test followed by post hoc Bonferroni correction (B), ANOVA and Tukey’s multiple comparison test (G).

Physiologically, IL-2 is a secreted molecule able to elicit long-distant effects. To better understand whether and how a membrane anchor would influence the function of membrane tethered IL-2, we inserted one, two or four Ig-like domains of CD16b between IL-2 and the minimal GPI-anchor attachment sequence of CD16b (see S1 Fig) [9]. Upon transfection, all IL-2 length variants (IL-2v) except IL-2::1IgGPI consistently resulted in more than 40% of IL-2 positive cells (Fig 1A and 1B). This was in contrast to the immunoblot analyses of whole cell lysates, in which IL-2::1IgGPI was expressed at similar levels to IL-2::GPI and IL-2::4IgGPI (Fig 1C). Intensity ratios of IL-2 and p30Gag revealed that IL-2::2IgGPI becomes targeted more efficiently to constant amounts of virus-like nanoparticles (VNP) induced by co-transfection of HEK-293 cells with MoMLV *gag-pol* (Fig 1D and 1E and S1 Fig). All four IL-2 variants were clearly targeted to the lipid raft enriched top fractions 2 and 3 of isopycnic sucrose gradients (Fig 1F) similar to the lipid raft marker molecule (CD59) [25] and as opposed to CD147, which is excluded from lipid rafts and locates to the bottom fractions 6–9 of gradients.

Of note, IL-2::1IgGPI and IL-2::4IgGPI massively interfered with particle production as determined by dot-blot analyses (Fig 1G) that is why they were excluded from further evaluations. Taken together biochemical analyses showed that the 2IgGPI-membrane anchor increased lipid raft- and VNP-targeting of IL-2 and did not interfere with particle production when compared to IL-2::GPI.

### The biological activity of VNP decorated with different IL-2 variants (IL-2v) is critically influenced by the membrane-anchor of IL-2

**Fig 2:**
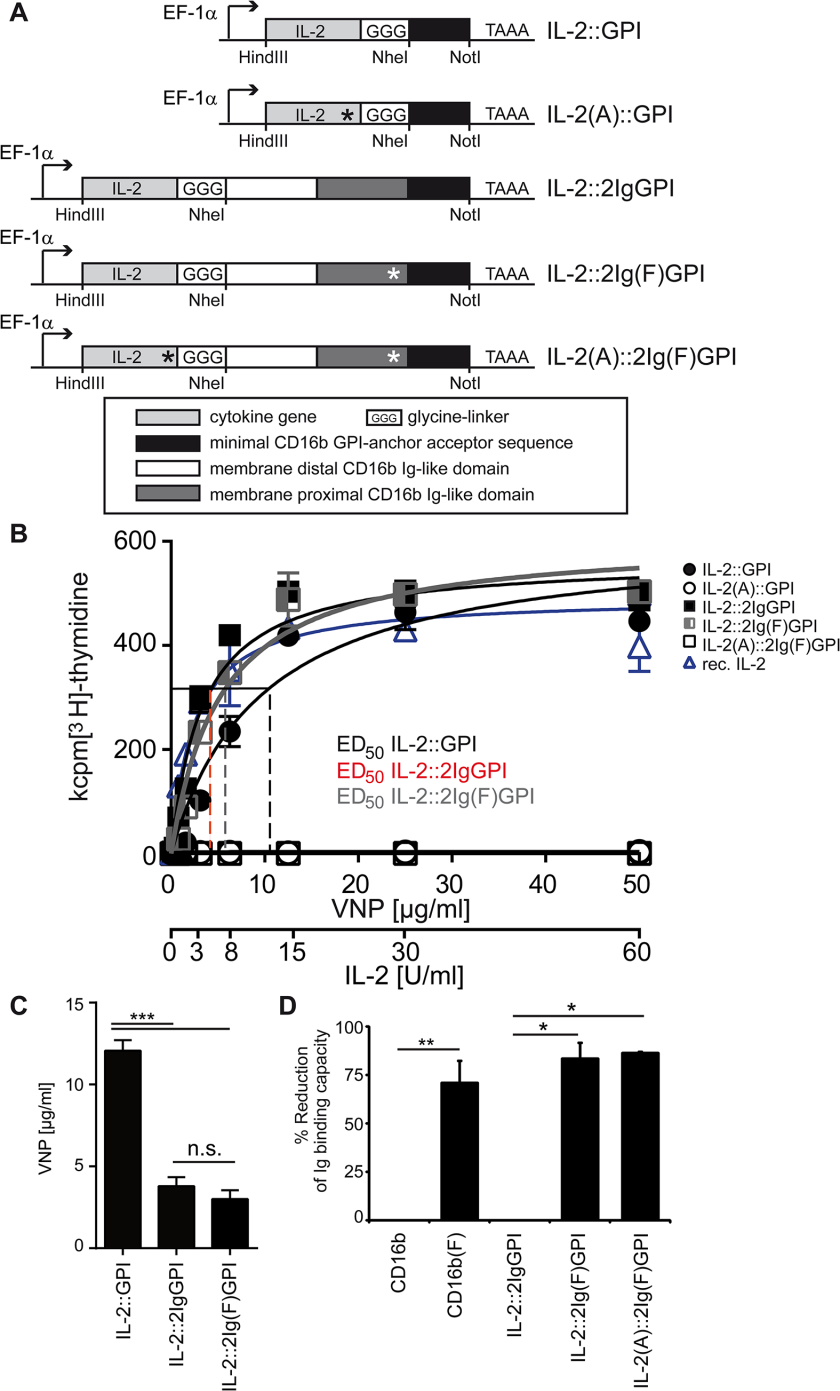
Scheme and functional evaluation of IL-2v. (**A**) Scheme of mutations introduced into IL-2v. IL-2 loss-of-function mutation (black asterisk), IgG-binding mutant (white asterisk). (**B**) Biological activity of IL-2v. IL-2 dependent HT-2 cells were incubated with titrated amounts of VNP decorated with either IL-2::GPI, IL-2::2IgGPI or their p.C72A mutated relatives IL-2(A)::GPI, IL-2::2Ig(F)GPI and IL-2(A)::2Ig(F)GPI, or alternatively recombinant IL-2 and analyzed for proliferation. ED_50_ values for IL-2::GPI (dashed black line), IL-2::2IgGPI (dashed red line), IL-2::2Ig(F)GPI (dashed blue line) decorated VNP are indicated. (**C**) Amounts of particles required to induce half maximal proliferation rates in HT-2 cells. (**D**) Flow cytometry analysis of IgG binding to HEK-293 cells transfected with indicated constructs. Figure shows percent reduction of Ig binding compared to unmodified molecules. Data are representative (B) or show the summary (C, D) of eleven (IL-2::GPI), seven (IL-2::2IgGPI) and six (IL-2::2Ig(F)GPI, IL-2(A)::GPI, IL-2(A)::2Ig(F)GPI and recombinant IL-2) (B), eleven (IL-2::GPI), seven (IL-2::2IgGPI) and six (IL-2::2Ig(F)GPI) (C) and five (except four IL-2(A)::2Ig(F)GPI) (D) independent experiments. * p < 0.05, **, p < 0.01, ***, p < 0.001; ANOVA and Tukey’s multiple comparison test (C) and Kruskal-Wallis test and Mann-Whitney U-test followed by post hoc Bonferroni correction (D).

To discern specific from non-specific IL-2 activity of VNP, loss of function versions of IL-2 were generated by mutating cysteine 72 to alanine (p.C72A) [22] (Fig 2A). To prevent IgG binding to the CD16-derived 2Ig moiety, and thus possible interference with the biological function of IL-2 or the half-life of VNP, position 112 of CD16b was mutated from tryptophan to phenylalanine (p.W112F) [23], leading to 70–90% reduced IgG binding capacity (Fig 2A and 2D and S2 Fig). These constructs were referred to as IL-2(A)::GPI, IL-2::2Ig(F)GPI, and IL-2(A)::2Ig(F)GPI, respectively (Fig 2A). The biological activity of membrane-anchored IL-2v was determined with the IL-2 dependent T cell line HT-2, which revealed that both IL-2::2IgGPI and IL-2::2Ig(F)GPI VNP are approximately 4-fold more effective than IL-2::GPI VNP (Fig 2B and 2C). These results also clearly prove that mutation of the IgG binding site in the IL-2::2IgGPI molecule does not alter its IL-2 dependent stimulatory capacity. VNP expressing the mutated IL-2v IL-2(A)::GPI and IL-2(A)::2Ig(F)GPI did not show any stimulatory activity (Fig 2B). Of note, there was comparable targeting to VNP of mutated and non-mutated molecules (see S2 Fig).

### Highly efficient activation and expansion of CD8^+^ T cells by IL-2::2Ig(F)GPI asVNP *in vitro*

**Fig 3:**
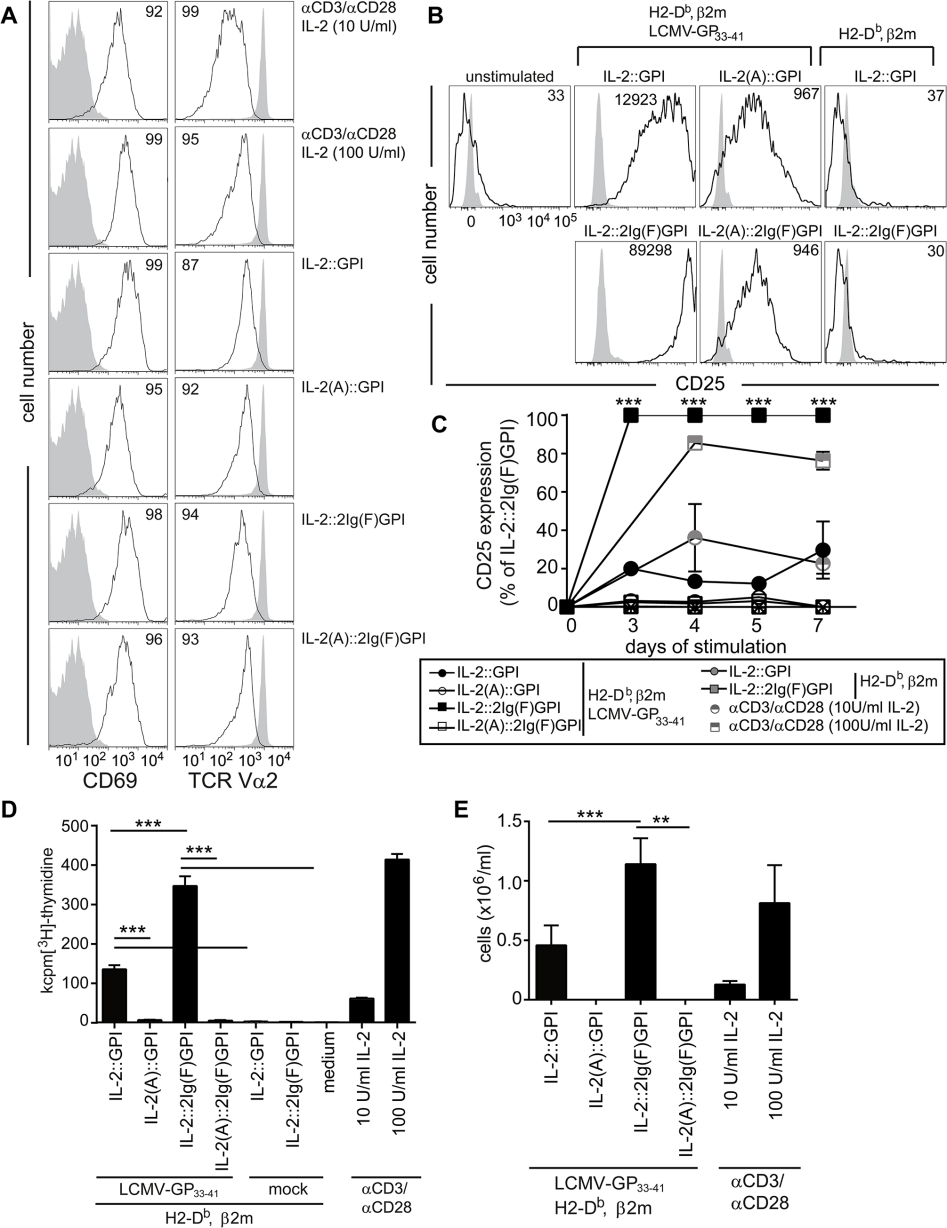
Differential activation of CD8^+^ T cells by IL-2v decorated VNP. (**A**) Flow cytometry analysis of CD69 and TCR expression on P14 TCR transgenic splenocytes. Cells were either left untreated (grey shaded histograms), or stimulated (black line) with IL-2v asVNP or alternatively with αCD3/αCD28 coated microbeads supplemented with 10 or 100 U/ml IL-2 for 18 hours. Percentages of CD69^**high**^ and TCR^**low**^ cells are shown. (**B** and **C**) Flow cytometry analysis of CD25 expression on CD8^**+**^ T cells. Purified P14 CD8^**+**^ T cells were stimulated for the indicated number of days with IL-2v asVNP, or αCD3/αCD28 coated microbeads supplemented with 10 or 100 U/ml IL-2, as indicated or were left untreated. (**B**) CD25 expression on CD8^**+**^ T cells co-cultured with IL-2v asVNP for three days. Numbers indicate MFI of CD25^**+**^ cells. (**C**) Kinetics of CD25 expression on CD8^**+**^ T cells stimulated with indicated asVNP, ansVNP, or αCD3/αCD28 coated microbeads supplemented with 10 or 100 U/ml IL-2. (**D**) Proliferation of CD8^**+**^ T cells upon incubation with IL-2v asVNP, ansVNP, medium or PMA/ionomycin. (**E**) Absolute numbers of viable P14 CD8^**+**^ T cells after 5 days of co-culture with IL-2v asVNP or αCD3/αCD28 coated microbeads supplemented with IL-2. Data are representative (A, B) or show the summary (C-E) of four (except two for αCD3/αCD28) (A), five (except three for ansVNP) (B), at least three (C), nine (except five for ansVNP and two αCD3/αCD28 microbeads) (D) and seven (except four for αCD3/αCD28 microbeads) (E) independent experiments. * p < 0.05, **, p < 0.01, ***, p < 0.001; t-test comparing IL-2::GPI with IL-2::2Ig(F)GPI asVNP (C), Kruskal-Wallis test and Mann-Whitney U-test followed by post hoc Bonferroni correction (D) ANOVA and Tukey’s multiple comparison test (E).

Prolonged IL-2R signals favor terminal-effector differentiation in CD8^+^ T cells [14] which is thought to depend on CD4^+^ T cell regulation of CD25 expression [29]. Since IL-2 regulates CD25 expression [30], we tested whether IL-2v expressing antigen-specific (as)VNP would differentially activate antigen-specific CD8^+^ T cells leading to differential up-regulation of CD25. For that purpose, we co-cultured purified P14 TCR transgenic CD8^+^ T cells with IL-2v asVNP for 18 hours. This led to homogeneous and significant neo-expression of CD69 on more than 90% and down-modulation of TCR surface expression on more than 70% of T cells, which was indicative of comparable TCR-specific signals provided by asVNP under investigation (Fig 3A). Next we tested neo-expression of the high-affinity IL-2 receptor (CD25) as a sign for sustained CD8^+^ T cell activation. We found significant up-regulation of CD25 (11±4-fold) on purified CD8^+^ T cells after 72 hours of co-culture with asVNP when compared to non-stimulated CD8^+^ T cells or CD8^+^ T cells co-incubated with ansVNP expressing functional IL-2 (Fig 3B and 3C). Decoration of asVNP with biologically active IL-2v led to a further up-regulation of CD25 by 23±13-fold (IL-2::GPI) or 70±18-fold (IL-2::2Ig(F)GPI), respectively. Significantly, CD8^+^ T cells stimulated with IL-2::2Ig(F)GPI maintained significantly higher CD25 expression from day 3 onwards when compared to IL-2::GPI asVNP (Fig 3C), which was comparable to cultures stimulated with αCD3/αCD28 coated microbeads and supplemented with low (10 U/ml) versus high (100 U/ml) dose IL-2.

IL-2v ansVNP and asVNP lacking biologically active IL-2v neither induced T cell proliferation nor survival beyond day 5 of culture (Fig 3D). In contrast, both IL-2::GPI and IL-2::2Ig(F)GPI asVNP induced significant T cell proliferation, with IL-2::2Ig(F)GPI asVNP being 3.1±0.1-fold more effective than IL-2::GPI asVNP. Thus, co-incubation with IL-2::GPI or IL-2::2Ig(F)GPI asVNP supports CD8^+^ T cell viability beyond day 5 of co-culture similar to αCD3/αCD28 coated microbeads supplemented with low (10 U/ml) or high (100 U/ml) dose of IL-2 (Fig 3E). Proliferation and expansion of antigen-specific CD8^+^ T cells beyond day 5 critically depends on the IL-2 signal received during early antigen-specific stimulation. IL-2::2Ig(F)GPI asVNP are clearly more efficient in providing this signal to T cells. These results are in contrast to previous findings showing that only IL-2 treatment in the ‘death phase’ (contraction phase), i.e. in the period 8 to 15 days after infection with specific virus, maintained higher cell numbers of virus-specific T cells while early IL-2 administration rather induced cell death [30].

### Terminally differentiated CD8^+^ T cells with high effector potential induced by IL-2::2Ig(F)GPI asVNP

**Fig 4:**
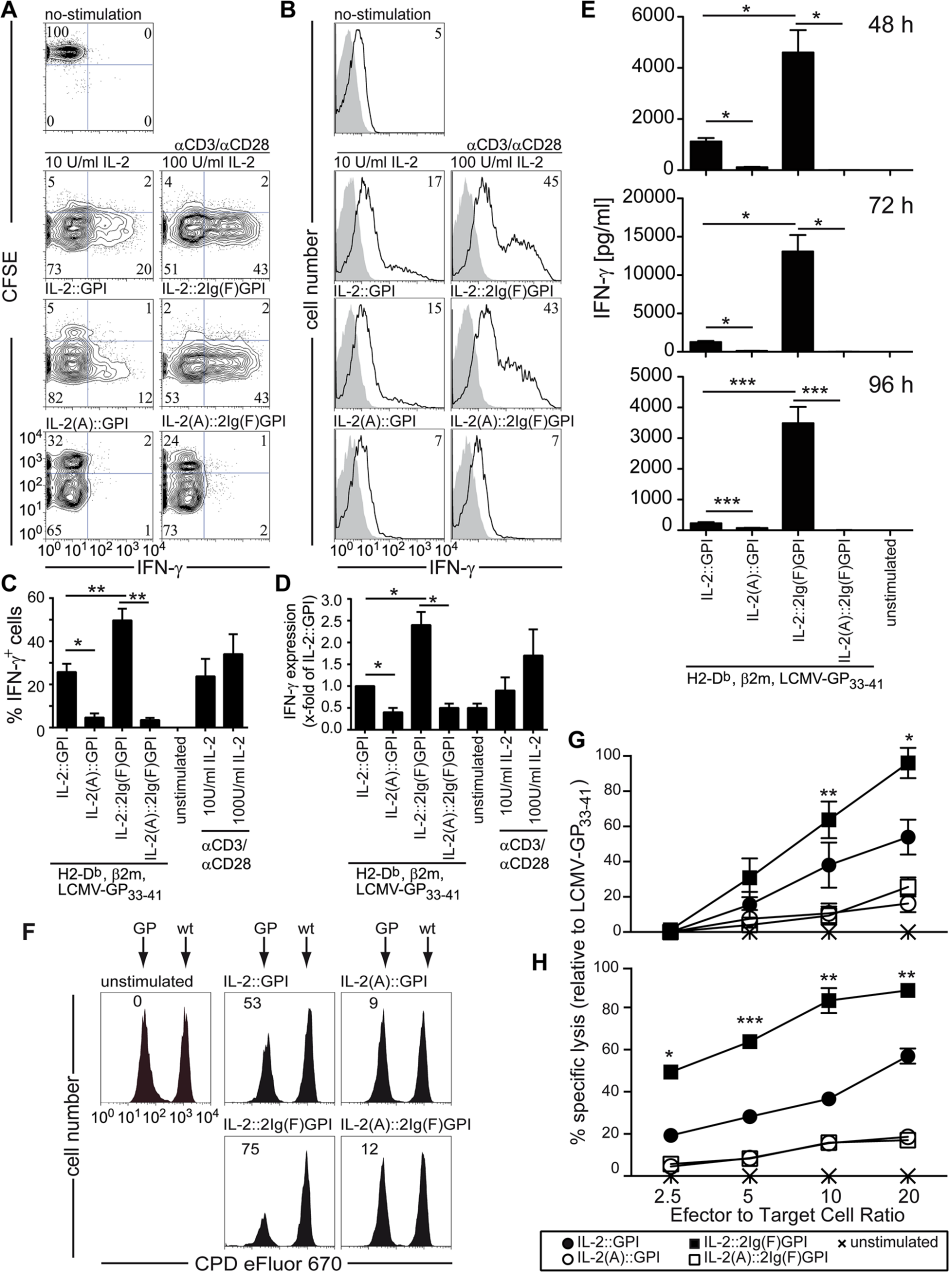
Strong CD8^+^ T cell effector functions induced by IL-2::2Ig(F)GPI asVNP. (**A**) Flow cytometry analysis showing proliferation and intracellular IFN-γ-expression of CFSE-labeled CD8^**+**^ TCR Vα2^**+**^ T-cells. Markers set according to negative stainings and no-stimulation control. Numbers indicate percentage of cells in respective quadrants. (**B**) Flow cytometry analysis, (**C**) percentage and (**D**) fold-induction of IFN-γ producing CD8^**+**^ T cells in the absence or presence of indicated stimuli. (**E**) Amounts of IFN-γ (pg/ml) secreted upon co-culture of CD8^**+**^ T cells with indicated IL-2v asVNP or αCD3/αCD28 coated microbeads plus IL-2 (10 or 100 U/ml). (**F-H**) IL-2::2Ig(F)GPI asVNP increase the cytotoxic potential of antigen-specific T cells. P14 splenocytes were stimulated with IL-2v asVNP for 48 hours or left untreated and were subsequently co-cultured with 1x10^**4**^ wt (CPD^**bright**^) or LCMV-GP expressing (CPD^**dim**^) EL-4 target cells. Specific target cell lysis was analyzed after 20 hours by flow cytometry. (**F**) Flow cytometry analysis showing specific lysis of LCMV-GP^**+**^ but not wt target cells at an effector/target ratio of 10. (**G**) Quantification by flow cytometric analysis of specific target cell lysis relative to maximum lysis (LCMV-GP_33-41_ stimulated effector cells, E:T ratio 20:1). (**H**) Determination of cytotoxic potential in ^**51**^Cr-release assays of P14 splenocytes, which were stimulated with IL-2v asVNP or left untreated for 48 hours. Effector cells were co-cultured with ^**51**^Cr-labelled wildtype or LCMV-GP-expressing EL-4 target cells (1x10^**4**^) and specific lysis was analyzed. Data are representative (A, B, F, H) or show the summary (C, D, E, G) of three (A, B, F, G), five (except three for αCD3/αCD28 microbeads) (C, D), four (except two for 48 and 72 hrs) (E) and triplicates of one (H) independent experiments. * p < 0.05, **, p < 0.01, ***, p < 0.001; ANOVA and Tukey’s multiple comparison test (C, D), Kruskal-Wallis test and Mann-Whitney U-test and Bonferroni correction (E), t-test comparing IL-2::GPI with IL-2::2Ig(F)GPI asVNP (G, H).

Next we investigated whether CD8^+^ T cells stimulated with the various asVNP display differential functional capabilities. In comparison to IL-2::GPI asVNP, IL-2::2Ig(F)GPI asVNP induced significantly higher percentages of IFN-γ^+^ TCR Vα2^+^CD8^+^ T cells while asVNP expressing the mutated IL-2 versions IL-2(A)::GPI or IL-2(A)::2Ig(F)GPI completely failed to do so (Fig 4A and 4C). The above results were mirrored by stimulating CD8^+^ T cells with αCD3/αCD8 coated microbeads in the presence of either low (10 U/ml) or high (100 U/ml) dose IL-2, respectively (Fig 4A and 4C). Moreover, the IFN-γ expression intensity of IL-2::2Ig(F)GPI compared to IL-2::GPI asVNP stimulated CD8^+^ T cells was significantly increased (Fig 4B and 4D). Similar results were obtained by stimulating whole splenocytes (see S3 Fig). Determination of secreted IFN-γ levels revealed even more significant differences between IL-2::2Ig(F)GPI and IL-2::GPI asVNP stimulated T cells and analyzed at multiple time points (Fig 4E). Significantly, CD8^-^ T cells in splenocyte cultures neither proliferated nor did they produce IFN-γ (see S3 Fig). Next we tested whether IL-2v asVNP increase the cytotoxic capability of virus-specific CD8^+^ T cells. For that purpose titrated amounts of differentially stimulated CD8^+^ T cells (CFSE^+^) were co-incubated with constant amounts of either antigen-specific, LCMV-GP expressing (CPD^dim^) or wild-type (CPD^bright^) EL-4 cells. In comparison to IL-2::GPI asVNP stimulated CD8^+^ T cells, IL-2::2Ig(F)GPI asVNP stimulated CD8^+^ T cells revealed a significantly higher cytotoxic potential (64±11 versus 38±13% target cell lysis) at an effector to target cell ratio of ten (Fig 4F). This was confirmed in dose-response experiments at all effector to target ratios tested (Fig 4G). These results were perfectly mirrored by CD107a (LAMP1) surface expression levels, showing the high degree of cytotoxic granule exocytosis (see S3 Fig). Results obtained by flow cytometry-based cytotoxicity assays were confirmed by classical chromium release assays (Fig 4H).

### CD8^+^ T cells acquire memory-like phenotype and function upon culture with IL-2v asVNP

**Fig 5:**
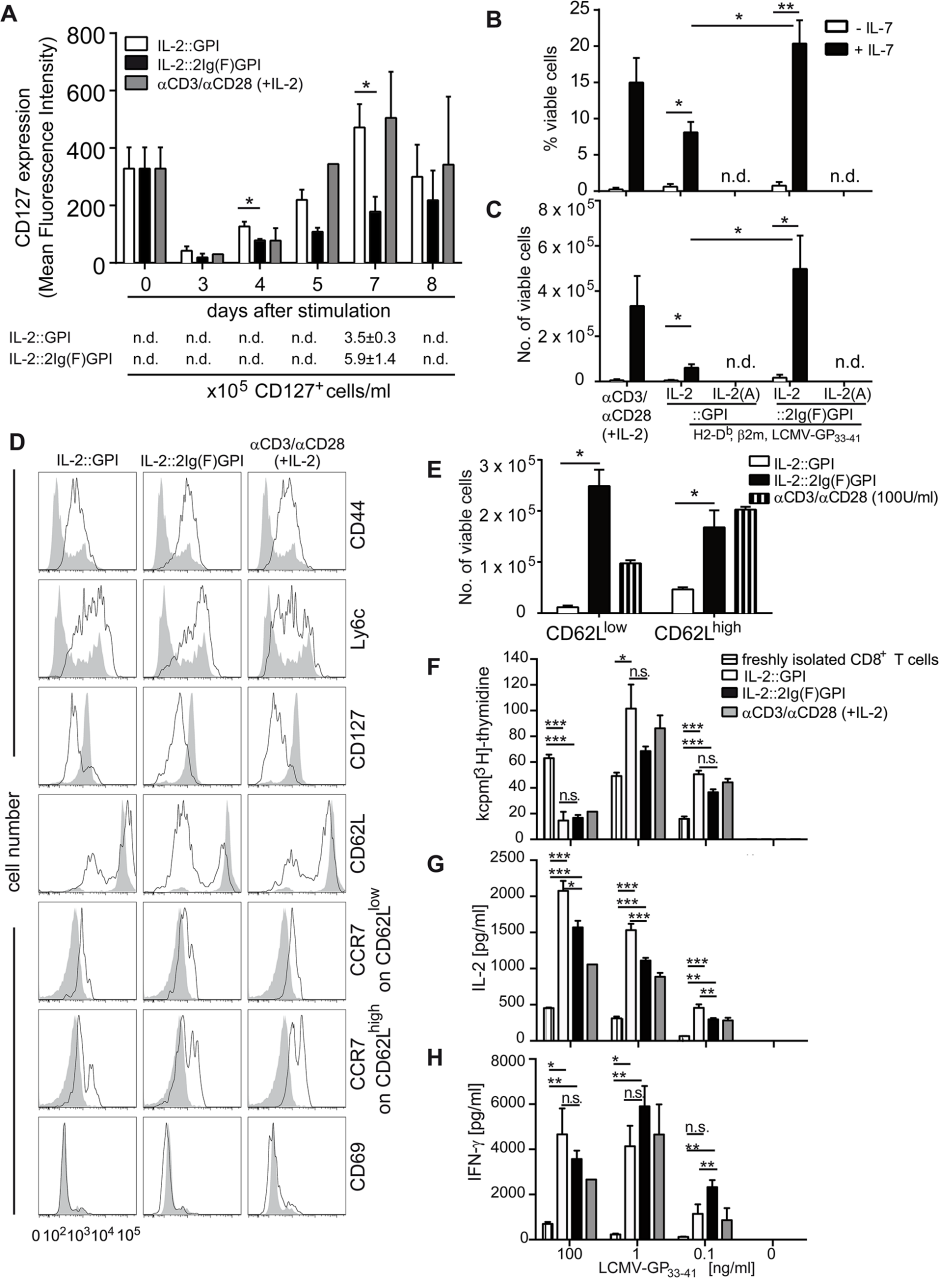
Acquisition of memory-like functions by CD8^+^ T cells is supported by IL-2 decorated asVNP. (**A**) Re-expression kinetics of CD127 on IL-2v asVNP stimulated purified P14 CD8^**+**^ TCR Vα2^**+**^ T cells. Mean fluorescence intensity of CD127 expression on CD8^**+**^ T cells at indicated time points after stimulation with IL-2::GPI, IL-2::2Ig(F)GPI asVNP or αCD3/αCD28 microbeads plus 100 U/ml IL-2. Bottom panel indicates absolute CD127^**+**^ cell numbers obtained at day 7. (**B**) Percentage and (**C**) absolute numbers of viable (propidium iodide negative) CD8^**+**^ T cells upon VNP-mediated primary stimulation for 7 days followed by removal of particles and a 4-day culture ±IL-7 (125 U/ml). n.d, not determinable (T cells did not survive day 7 of primary culture). (**D**) Flow cytometry analysis showing memory and activation phenotype of naïve (shaded grey histograms) and IL-2v asVNP or αCD3/αCD28 microbeads plus 100 U/ml IL-2 activated cells (black line). (**E**) Absolute numbers of viable CD8^**+**^CD25^**+**^CD62L^**low**^ and CD8^**+**^CD25^**+**^CD62L^**high**^ cells after 11 days of culture (co-culture and resting phase). n.d, not determinable (T cells did not survive day 7 of primary culture). (**F—H**) Secondary responses of CD8^**+**^ T cells upon pre-stimulation with IL-2v. Viable cells (1x10^**4**^) pre-stimulated as indicated were re-stimulated with LCMV-GP_33-41_ peptide pulsed or non-pulsed irradiated splenocytes. Freshly isolated CD8^**+**^ T cells served as control. (**F**) Proliferation was determined by [^**3**^H]-thymidine incorporation after 48 hours of stimulation. Freshly purified CD8^**+**^ T cells served as control. Secreted (**G**) IL-2 and (**H**) IFN-γ levels in supernatants of CD8^**+**^ T cells during secondary responses. Data are representative (D) or show the summary (A, B, C, E-H) of three (except two for αCD3/αCD28 microbeads plus IL-2) (A), five (except three for αCD3/αCD28 microbeads plus IL-2) (B, C), three (D-H) independent experiments. * p < 0.05, **, p < 0.01, ***, p < 0.001; t-test (A, E), ANOVA and Tukey’s multiple comparison test (B, F-H), Kruskal-Wallis test and Mann-Whitney U-test and Bonferroni correction (C).

Previous studies indicated that earlier and sustained re-expression of CD127 upon antigenic stimulation of CD8^+^ effector cells is associated with their differentiation into memory cells [31–35]. In fact, after initial down-regulation of CD127 IL-2::GPI or IL-2::2Ig(F)GPI asVNP co-cultured CD8^+^ T cells started to re-express CD127 from day 4 onwards (Fig 5A and S4 Fig). Interestingly, re-expression of CD127 on CD8^+^ T cells obtained from IL-2::2Ig(F)GPI asVNP stimulated cultures was significantly retarded until day 7 of culture when compared to IL-2::GPI asVNP (Fig 5A and S4 Fig). Co-culture of CD8^+^ T cells with αCD3/αCD28 coated microbeads supplemented with high dose IL-2 (100 U/ml IL-2) mirrored CD127 re-expression kinetics of IL-2::GPI asVNP stimulated CD8^+^ T cells, while αCD3/αCD28 coated microbeads supplemented with low dose IL-2 (10 U/ml) did not support cell viability beyond day 5 of culture. This suggests that soluble IL-2 added as a single dose to such cultures might have a lower bioavailability when compared to a single dose of VNP-bound IL-2. In comparison to IL-2::2Ig(F)GPI expanded T cells, IL-2::GPI expanded T cells contained a higher percentage of memory precursor-like CD127^+^CD8^+^ T cells on day 7. However, in comparison to IL-2::GPI asVNP, IL-2::2Ig(F)GPI asVNP expanded CD8^+^ T cells more vigorously and therefore, in absolute terms, generated 1.7±0.3-fold more memory precursor-like CD8^+^ T cells re-expressing CD127. Control cultures performed with IL-2v ansVNP or mutated IL-2v asVNP did neither support CD127 modulation nor survival (Fig 3 and S4 Fig), respectively.

Next, we investigated whether IL-2v asVNP differentially shape the CD8^+^ T cell memory-like phenotype and function *in vitro*. Therefore, we stimulated purified P14 TCR transgenic CD8^+^ T cells with IL-2v asVNP for 7 days, and re-cultured them for 4 days in the presence or absence of the pro-survival factor IL-7 [36]. CD8^+^ T cells stimulated with asVNP in the absence of IL-2 and those stimulated with αCD3/αCD28 coated microbeads in the presence of low dose IL-2 (10 U/ml) did not survive beyond day 7 after initial stimulation and could therefore not be further analyzed. Moreover, IL-7 was found to be critically required during the 4-day resting period since only very low numbers of viable cells (< 1%) could be rescued in the absence of IL-7 (Fig 5B and 5C). Both the percentage and number of viable cells was larger in IL-2::2Ig(F)GPI pre-cultured cells and was comparable to αCD3/αCD28 coated microbeads plus 100 U/ml IL-2 co-cultured T cells (Fig 5B and 5C). This result does not directly correlate with CD127 expression levels, which are higher on IL-2::GPI asVNP pre-stimulated T cells and might indicate lower fitness of such cells or the requirement of additional survival signal(s) at this point.

Of note, the memory markers CD44 [37] and Ly6c [38] were clearly up-regulated on expanded CD8^+^ T cells irrespective of the primary stimulus applied (Fig 5D). CD127 expression was found negative, which is due to down-regulation induced by IL-7 supplementation of media during the resting period [39]. Freshly isolated CD8^+^ T cells displayed high levels of CD62L surface expression (Fig 5D). Co-culture of CD8^+^ T cells with IL-2::GPI asVNP generated a mixed population of cells with 19.5±9.6% CD62L^low^ and 78.7±12.7% CD62L^high^ cell, respectively. In contrast, IL-2::2Ig(F)GPI asVNP induced a significantly larger proportion of CD62L^low^ CD8^+^ T cells (59.5±13.2%) resulting in significantly less CD62L^high^ CD8^+^ T cells (40.1±13.8%) (Fig 5D). Interestingly, T cells stimulated with αCD3/αCD28 microbeads in the presence of high dose IL-2 (100 U/ml) generated proportions of CD62L^high^/CD62L^low^ cells similar to IL-2::GPI asVNP (Fig 5D).

In comparison to CD62L^low^ cells, CD62L^high^ T cells expressed significantly higher levels of the chemokine receptor CCR7, which is another hallmark of central memory T cells [40]. Thus, when compared to IL-2::GPI asVNP, IL-2::2Ig(F)GPI asVNP in the presence of IL-7 (125 U/ml) promoted the development of approximately 24-fold more CD62L^low^CCR7^low^CD8^+^ effector memory-like T cells after the 11-day culture period while 4-fold more CD62L^high^CCR7^high^CD8^+^ central memory-like T cells were generated (Fig 5E). Significantly, the clear-cut CD69 expression observed after 18 hours of stimulation (Fig 3A) has returned to background levels on day 11, indicating that the CD8^+^ T cells, indeed, returned to a resting state.

To evaluate their memory-like function we re-stimulated equal numbers of viable CD8^+^ T cells obtained after the 11-day culture period with irradiated splenocytes from C57/BL6 wildtype mice, pulsed with titrated amounts of antigen-specific peptide. While freshly isolated CD8^+^ T cells proliferated less with decreasing amounts of antigen (Fig 5F), CD8^+^ T cells obtained from asVNP cultures responded weaker to high (100 ng/ml) but significantly stronger to low (0.1 ng/ml) amounts of antigen (p<0.001) (Fig 5F). Of note, CD8^+^ T cells did not proliferate in the absence of antigen irrespective, which IL-2v was expressed on asVNP during primary stimulation (Fig 5F). However, in the secondary response we did not observe differences regarding proliferation of CD8^+^ T cells obtained from IL-2::GPI, IL-2::2Ig(F)GPI asVNP or αCD3/αCD28 microbeads plus IL-2 stimulated cultures (Fig 5F). Therefore, CD8^+^ T cells, which had been pre-stimulated with IL-2v asVNP, displayed a memory-like hyper-proliferative phenotype upon secondary stimulation especially when antigen was limited. Consistent with their memory-like phenotype, pre-activated CD8^+^ T cells produced significantly more IL-2 and IFN-γ at all antigen concentrations evaluated when compared to freshly isolated CD8^+^ T cells (Fig 5G and 5H). CD8^+^ T cells incubated with IL-2::2Ig(F)GPI asVNP during primary stimulation secreted significantly less IL-2 but much more IFN-γ during the secondary response when stimulated with low amounts of antigen (0.1 ng/ml) when compared to IL-2::GPI asVNP stimulated cells. These results are compatible with Blimp-1-mediated repression of IL-2 production [41, 42]. While the type of primary stimulation did not influence the degree of proliferation during the secondary response it significantly influenced the secreted cytokine profile during secondary stimulation. Taken together, IL-2::GPI asVNP favored IL-2 secreting cells compatible with a central memory-like phenotype while IL-2::2Ig(F)GPI asVNP promoted IFN-γ producing effector memory-like cells.

### Induction of effector functions and memory phenotypes by IL-2v asVNP

**Fig 6:**
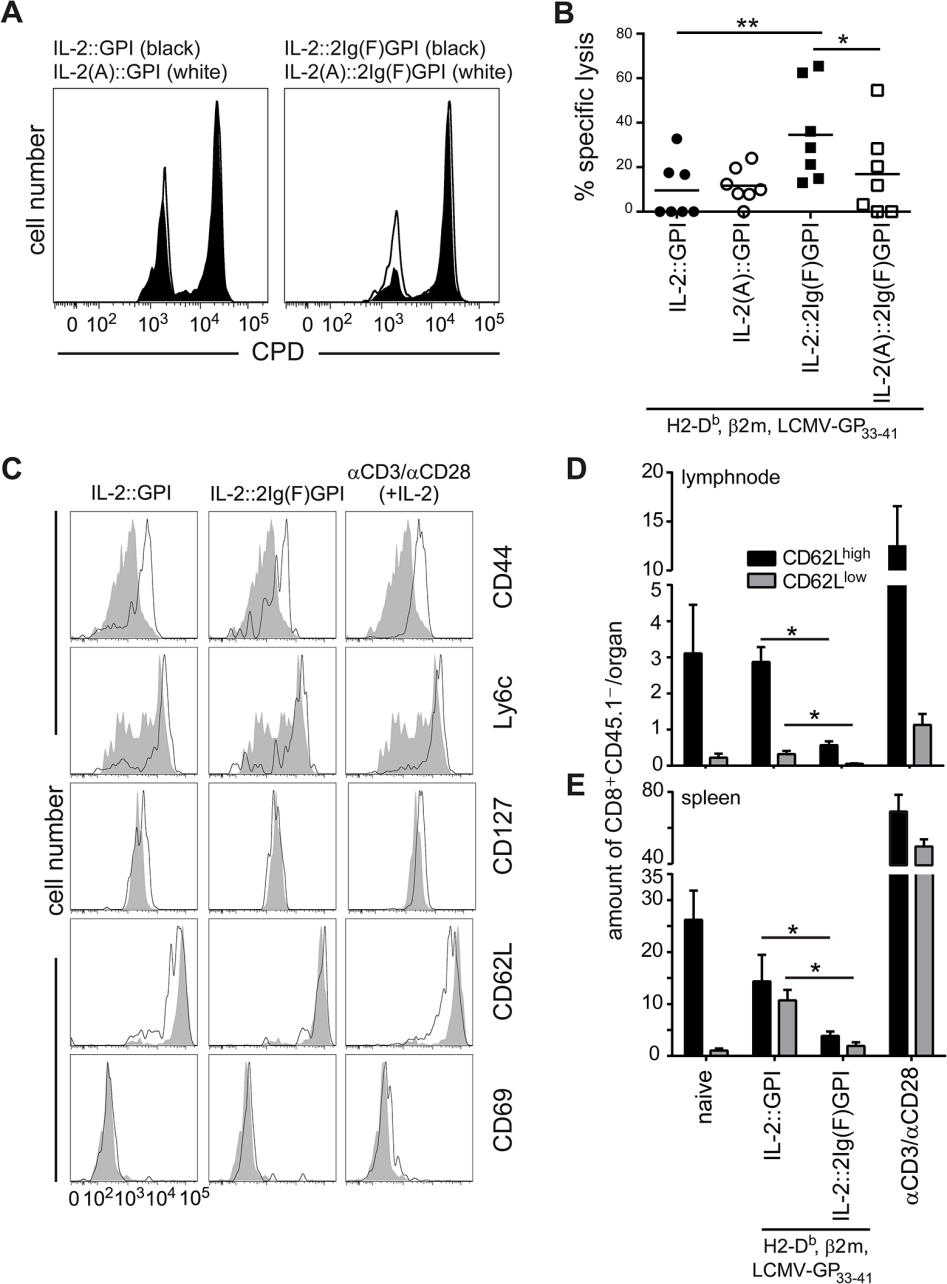
Induction of effector functions and memory phenotypes by IL-2v asVNP. Enhanced *in vivo* cytotoxicity of CD8^**+**^ effector T cells in mice immunized with IL-2::2Ig(F)GPI asVNP. (**A**) Flow cytometry analysis showing recovery of adoptively transferred and differentially CPD labeled LCMV-GP_33-41_ peptide (CPD^**dim**^) or mock peptide (CPD^**bright**^) pulsed congenic CD45.1^**+**^ splenocytes from CD45.2^**+**^ wild-type recipient mice. Two days before target cell transfer, wt mice received 2x10^**5**^ purified P14 CD45.1^**+**^CD8^**+**^ effector T cells and were immunized with IL-2::GPI or IL-2::2Ig(F)GPI asVNP (black histograms) or with IL-2(A)::GPI or IL-2(A)::2Ig(F)GPI asVNP (open histograms) or were left untreated. (**B**) Quantification of specific target cell lysis *in vivo*. Horizontal lines indicate the mean. (**C-E**) Pre-stimulation of CD8^**+**^ T cells with IL-2::GPI asVNP generates higher absolute numbers of memory cells *in vivo*. P14 CD8^**+**^ T cells (CD45.2^**+**^) were *in vitro* pre-stimulated with IL-2v asVNP or αCD3/αCD28 microbeads (+100 U/ml IL-2) for six days. Equal amounts of viable naïve and activated cells (1x10^**6**^) were adoptively transferred into CD45.1^**+**^ congenic recipient mice. After 7 days engraftment of P14 CD8^**+**^CD45.2^**+**^ T cells in the inguinal lymphnodes and the spleens were determined by flow cytometry. (**C**) Flow cytometry analysis showing expression of the indicated markers on naïve (shaded grey histograms) and *in vitro* pre-activated (black line) CD8^**+**^CD45.2^**+**^ donor cells isolated from the lymph nodes of recipient mice. Absolute numbers of CD62L^**low**^ and CD62L^**high**^ donor cells recovered from (**D**) inguinal lymphnodes and (**E**) spleens of recipient mice. Data are representative (A, C) or show the summary (B, D, E) of 28 mice (seven per group) that were analyzed in four independent experiments (A, B), or of 18 mice (eight per group, except for naïve (two), IL-2::GPI (three), αCD3/αCD28 microbeads plus IL-2 (five)) (C-E) that were analyzed in two independent experiments. * p < 0.05, **, p < 0.01 ANOVA, t-test and post hoc Bonferroni correction (B); Mann-Whitney U-test (D, E).

Next we tested the potential of IL-2v asVNP to instruct CD8^+^ T cells for short-term effector functions *in vivo*. We adoptively transferred purified P14 CD45.2^+^ CD8^+^ T cells into C57BL/6 CD45.2^+^ wild-type recipient mice and immunized them with IL-2::GPI, IL-2(A)::GPI, IL-2::2Ig(F)GPI and IL-2(A)::2Ig(F)GPI asVNP or PBS. Two days after immunization we transferred differentially CPD labeled LCMV-GP_33-41_ peptide (CPD^dim^) or mock peptide (CPD^bright^) pulsed splenocytes obtained from congenic CD45.1^+^ donor mice as target cells into the CD45.2^+^ recipient mice and analyzed specific target cell lysis after another two days by flow cytometry. We found that IL-2::2Ig(F)GPI asVNP are significantly superior (34.5±8.2% target cell lysis) to all other asVNP (< 17% lysis) tested in respect to their ability to instruct CD8^+^ T cells for cytotoxic lysis of specific target cells *in vivo* (Fig 6A and 6B). No differences in specific target cell lysis were observed between the groups of mice immunized with IL-2::GPI and IL-2(A)::GPI asVNP. This might indicate that the cellular activation brought about by signal one alone overshadows the contribution of the IL-2::GPI co-stimulus, which is otherwise clearly demonstrable in *in vitro* experiments. In summary, CD8^+^ T cell effector functions can be modulated also *in vivo* by membrane bound IL-2 attached to optimal membrane anchors applied as natural adjuvant expressed on asVNP.

Finally, we determined whether congenic adoptively transferred CD8^+^ T cells differentiated *in vitro* by IL-2v asVNP or αCD3/αCD28 microbeads plus IL-2 give also rise to T cell populations with a memory phenotype *in vivo*. For that purpose, the total number of antigen-specific memory T cells with the phenotype CD44^high^Ly6c^high^CD127^high^CD69^neg^ co-expressing either CD62L^high^ or CD62L^low^ (Fig 5C and S4 Fig) recovered 7 days after transfer from lymph nodes and spleen were determined (Fig 6C, 6D and 6E and S4 Fig). As expected, lymph nodes almost exclusively contained CD62L^high^ memory T cells. Of note, similar numbers of CD62L^high^ and CD62L^low^ memory T cells were recovered from spleens of recipient mice. In contrast to the IL-2v asVNP pre-stimulated T cells, transferred naïve T cells used as control also homed to lymph nodes and spleens but did not reveal any signs of a memory phenotype as determined by flow cytometry (Fig 6C and S4 Fig). Significantly, absolute numbers of recovered T_CM_ and T_EM_ were much higher upon adoptive transfer of IL-2::GPI asVNP as compared to IL-2::2Ig(F)GPI asVNP pre-stimulated CD8^+^ T cells (Fig 6D and 6E). The significantly lower numbers of T cells recovered upon adoptive transfer of IL-2::2Ig(F)GPI asVNP stimulated CD8^+^ T cells is indicative of a high number of short-lived effector T cells lacking memory potential in these cultures, which might not survive the *in vivo* resting phase of 7 days. However, and as describe above (Figs 3E and 5C), IL-2::2Ig(F)GPI asVNP expanded CD8^+^ T cells much more efficiently leading to significantly higher absolute cell numbers after 5, 7 and 11 days of *in vitro* cultures. CD8^+^ T cells pre stimulated with αCD3/αCD28 microbeads and IL-2 resulted in the highest numbers of memory cells recovered, which might be due to the dual co-stimulus delivered via CD28 and the IL-2R.

## Discussion

Cytotoxic CD8^+^ T lymphocytes belong to the adaptive branch of the immune system and are stimulated by intracellular pathogens (viruses, intracellular bacteria) but also tumor antigens. IL-2 represents an important growth and differentiation factor for human T lymphocytes, which is especially relevant to shape CD8^+^ T cell fate and function. Lack of IL-2 leads to virus-induced overt lymphoproliferative disease [43] and autoimmunity [44], which, depending on the genetic background, manifests itself primarily as inflammatory bowel disease or hemolytic anemia [45]. On the other hand, high-dose intravenous administration of IL-2, e.g. for treatment of malignant melanoma and metastatic renal carcinoma, frequently causes severe adverse reactions dominated by capillary leak syndrome, hypovolemia and accumulation of fluid in the extra-vascular space along with organ failure [46], which is caused by an IFN-γ and TNF-α dominated ‘cytokine storm’ and thus limits its clinical use [47].

One strategy to directly target CD8^+^ T lymphocytes and to circumvent the above-described toxicity problems might be to locally restrict the action of IL-2 to the site of antigen-presentation, e.g. by co-localization of IL-2 with antigen-specific pMHC on a particulate platform. Along those lines we have developed a modular platform based on VNP, which can be conveniently decorated with pMHC and other immunomodulators of choice [9]. To modulate CD8^+^ T cell fate and function, different forms of membrane-bound IL-2 were developed and tested herein, among them also non-functional versions of IL-2 used as stringent controls. We demonstrate, that the quality of the IL-2 membrane anchor used to tether IL-2 to the VNP surface determines the degree of targeting of IL-2 and thus the co-stimulatory potency of IL-2 on antigen-specific VNP. Out of a collection of four different membrane anchors, differing in their anchor dimensions, the version relying on the insertion of two Ig-like domains between the minimal GPI-anchor acceptor sequence and the IL-2 molecule proved to be superior over the other candidates in terms of its i) degree of targeting to the VNP surface, ii) biological activity, iii) co-stimulatory potency, iv) induction of differentiation and acquisition of CD8^+^ T cell effector functions *in vitro* and *in vivo*. In contrast, the GPI version rather favored memory precursor cell formation as confirmed by adoptive transfer and *in vivo* resting experiments.

While the IL-2 expressing VNP described in this report potently activated cognate CD8^+^ T cells for proliferation and IFN-γ production, the same particles were unable to activate non-cognate T cells, such as the CD4^+^ T cells present in splenocyte VNP co-cultures (see S3 Fig). This indicates that in contrast to soluble IL-2, membrane-bound IL-2 provided by decorated VNP is unable to entertain bystander activation and thus elicit dreadful systemic toxicity, which clearly distinguishes VNP-bound IL-2 from its soluble relative and makes it a very interesting adjuvant candidate. IL-2 and pMHC have to become displayed on the same VNP since we have shown in a previous report, that asVNP supplemented with soluble IL-2 do not support the same degree of antigen-specific activation *in vivo* [10]. While short-term control experiments performed with αCD3/αCD28 microbeads supplemented with low (10 U/ml) or high (100 U/ml) dose IL-2 recapitulated in some systems (e.g. IFN-γ expression, antigen-specific proliferation) the effects of co-culture with IL-2::GPI and IL-2::2IgGPI asVNP, respectively, such correlations were, however, not observable for prolonged periods of culture. For instance, CD127 neo-expression induced by αCD3/αCD28 microbeads supplemented with high dose IL-2 (100 U/ml) follows kinetics similar to T cells co-cultured with IL-2::GPI asVNP. Thus, questions of bioavailability and stability together with possible improved accessibility to the T cells of IL-2v might be factors influencing the functional capabilities of the different membrane-bound IL-2 versions studied in this report.

One of the early events upon TCR-mediated activation of CD8^+^ T cells is the down-modulation of TCR surface expression [48] and the up-regulation of the activation marker CD69 [49, 50]. This is followed by up-regulation of the high-affinity IL-2 receptor CD25 and the initiation of rapid cellular proliferation. During this phase of expansion CD8^+^ T cells further differentiate and start to acquire effector functions. In agreement with previous reports we here demonstrate that the early events of CD8^+^ T cell activation (TCR down-modulation and CD69 neo-expression) are primarily modulated by antigen-specific pMHC and are largely independent of co-stimuli, in our case co-expressed IL-2 variants. However, clear-cut differences between the IL-2 variants evaluated were observed during later events of CD8^+^ T cell activation such as for example the up-regulation of the high-affinity IL-2R, CD25. While asVNP up-regulated CD25 expression moderately, IL-2v asVNP super-induced CD25 expression. Of note, IL-2::2Ig(F)GPI asVNP promoted a much stronger and more sustained up-regulation of CD25 when compared to IL-2::GPI asVNP. In accordance with these data, up-regulation of CD25 was detected in a previous study in LCMV infected mice upon exogenous IL-2 administration. In that study CD25 up-regulation was only observed when administered during the ‘death phase’ (contraction phase) of the anti-viral immune response, i.e. from day 8 to 15 [30]. Importantly, the level of early CD25 up-regulation during an immune response seems to be a decisive factor for the further differentiation steps of CD8^+^ T cells. In fact, CD25^high^ expressing CD8^+^ T cells have been shown to preferentially differentiate into IL-7R^low^ (CD127^low^) short-lived effector cells at later stages of CD8^+^ T cell development [14, 29, 51], producing high levels of cytotoxic effector molecules [14]. In contrast, CD25^low^ expressing cells preferentially differentiate into IL-7R^high^ (CD127^high^) memory precursor effector cells [29]. The IL-7R (CD127) status seems to be crucial for the survival of memory precursor effector cells since other important homeostatic factors relevant for CD8^+^ T cells, among them IL-15, were shown to be unable to replace IL-7 [52]. Our results obtained with the IL-2::GPI variant are fully compatible with previous reports showing that ‘weak IL-2 receptor signals’ induced either by lack of CD25 expression [29] or by blockade of IL-2 signaling with anti-IL-2 and-CD25 antibodies [53] favor memory cell proliferation and expansion. Moreover, the stronger IL-2R signal provided by our IL-2::2Ig(F)GPI variant expanded significantly more short-lived effector cells.

The most plausible molecular mechanism operative is an IL-2R-dependent shift in the balance between the two important transcriptional regulators B lymphocyte-induced maturation protein 1 (Blimp-1) and B-cell lymphoma 6 protein (Bcl-6) [54]. In fact, IL-2R signaling or extensive antigen stimulation induces Blimp-1, which promotes terminal effector cell differentiation, down-regulates CD127 (IL-7R α-chain) expression [32, 34] and IL-2 production and suppresses various other aspects of the transcriptional program necessary for memory cell formation either directly or via repression of Bcl-6 [14, 55]. In contrast, low IL-2R signaling favors the expression of Bcl-6 and reciprocally represses Blimp-1. It will be interesting to investigate this matter in more detail in future studies.

*Schoenberger* and collaborators have very elegantly shown that autocrine IL-2 production by CD8^+^ T cells suffices to expand CD8^+^ memory cells [18]. According to their model, CD4^+^ T cell help is directed towards APC, enabling them to up-regulate CD70, which ligates CD27 on CD8^+^ T cells and eventually transmits the obtained helper signal enabling CD8^+^ T cells to autocrine IL-2 production. By providing cognate antigen in the context of membrane-bound IL-2 as ‘helper factor’ for CD8^+^ T cells our particles combine functions of activated APC and helper T cells. We here show that this combination is able to ‘imprint’ CD8^+^ T cells with the ability to activation, differentiation and expansion. Whether this entails CD8^+^ T cells also for autocrine IL-2 production remains to be demonstrated in future studies.

Do the cells generated herein with the help of asVNP have the principle potential to develop into long-lived memory cells? Long-lived central memory cells are characterized by high-levels of CD62L expression (CD62L^high^), which endows them with the ability to preferentially migrate and reside in secondary lymphoid tissues [40]. Upon antigenic re-stimulation T_CM_ cells typically release large amounts of IL-2 while exhibiting only little effector functions. In contrast, CD62L^low^ effector-memory T_EM_ reveal a high potential for rapid proliferation and exert effector functions such as IFN-γ and TNF-α secretion and cytotoxic activity upon antigenic stimulation. Our *in vivo* studies confirm the memory inducing potential of IL-2v asVNP. CD8^+^ T cells pre-activated with IL-2::GPI decorated VNP gave rise to both CD62L^high^ and CD62L^low^ spleen-homing lymphocytes. In contrast, IL-2::2Ig(F)GPI asVNP pre-stimulated T cells also gave rise to T_CM_ cells, however, at a much lower frequency. This was compatible with the much lower percentage of memory-like precursor cells in such cultures. The fact that no reciprocal increase in T_EM_ cells was observable upon transfer of IL-2::2Ig(F)GPI asVNP pre-stimulated cells is indicative for the expansion by IL-2::2Ig(F)GPI asVNP of short-lived effector cells lacking *bona fide* memory function rather than effector memory-like cells. Consequently, asVNP equipped with IL-2v represent an efficient novel tool to activate, expand and differentiate CD8^+^ T cells either more towards short-lived effector cells or rather towards memory precursor cells.

How could a potential therapeutic application of asVNP expressing membrane-bound IL-2 look like? The approach described here could be used to initiate a first wave of an antigen-specific immune response, e.g. against neoplastic or virally infected cell types. We have tested this hypothesis *in vitro* and *in vivo* and could, indeed, show that both neoplastic cells expressing a viral model antigen on the cell surface as well as splenocytes pulsed with an immunogenic viral peptide become much more efficiently eliminated by CD8^+^ T cells which had been instructed by IL-2::2Ig(F)GPI than those activated by IL-2::GPI asVNP. The high degree of specificity provided by the asVNP is probably best demonstrated by the lack of activation of CD8^-^ T cells in splenocyte cultures, which neither proliferated nor produced any IFN-γ (see S3 Fig). This qualifies the IL-2::2Ig(F)GPI asVNP platform, in principle, for quick induction of protective immunity in the naïve host. In principle, the multivalency of the VNP platform and its modular nature might be suitable to further refinements allowing, e.g. the introduction of additional antigens and/or immuno-modulators [9, 28]. Given the results obtained with αCD3/αCD28 microbeads, CD80 would be a very interesting candidate. Our results demonstrating vigorous proliferation of CD8^+^ T cells upon co-culture with IL-2v asVNP are somewhat in contrast to previous findings, which had shown that IL-2 treatment during the initiation of an immune response leads to systemic cytopenia with reduced spleen cell numbers and thymic involution [29]. In that study the authors argued that activation-induced cell death of effector cells was the major reason for this phenomenon. Interestingly, this does not seem to play a major role when virus-specific P14 T cells are co-stimulated with membrane bound IL-2 displayed by asVNP.

Is the finding that variation of the membrane anchors of membrane-bound IL-2 influences the biological activity of IL-2 unexpected? In fact, it has been demonstrated previously that the dimensions of *bona fide* cell surface molecules can have a decisive impact on their function especially in situations in which a coordinated interaction of several receptors, all embedded into the same biological membrane, is required [56]. Along those lines *Choudhuri et al*. have shown that elongation of the pMHC ectodomain greatly reduces TCR triggering without affecting TCR-pMHC ligation [56]. It was concluded that increased inter-membrane separation distances diminish segregation of larger molecules, such as CD45 [57], from the central supramolecular activation cluster of the immunological synapse, leading to high CD45-dependent tyrosine phosphatase activity which is incompatible with sustained TCR signaling. Since cytokines usually represent soluble molecules no *a priori* information as to their evolutionary-selected optimal membrane anchor dimensions was available. Usually, minimal GPI-anchor acceptor sequences with a length ranging from 22 to 26 amino acids are used to GPI-anchor molecules of choice to the plasma membrane [9, 10, 28]. Although we and others have shown that association of immuno-modulatory molecules fused to minimal GPI-anchors endows antigen presenting cells and VNP deriving from them with the specific biological activity of the respective human and murine molecules *in vitro* and *in vivo* [10] it remained unclear, especially for cytokines, whether the initially chosen minimal anchor dimensions would endow them with optimal biological function.

In conclusion, we here demonstrate, that the requirements for differential biological activities of artificially membrane-anchored cytokines decorating VNP used as convenient immunization platform cannot be predicted *a priori* but have to be evaluated *in vitro* and *in vivo* with scrutiny. Our studies add context and quality of the IL-2R signal as important additional parameters influencing IL-2 function to the already well-described factors such as timing of administration, differentiation state of responding T cells, and presence of antigen.

## Supporting Information

S1 Fig Illustration of the production of cytokine decorated VNP and IL-2v fused to different membrane anchors. (**A**) Strategies for anchoring IL-2 to the plasma membrane. (**B**) Cytokines genetically fused to membrane anchors containing GPI-anchor acceptor sequences, are—upon expression in HEK-293 producer cells—targeted to lipid rafts of the plasma membrane. Formation of plasma membrane derived VNP is induced by co-transfection of producer cells with MoMLV*gag-pol* (OGP). Lipid raft resident molecules are incorporated into the VNP arising. In order to activate or modulate target cells, VNP-containing HEK-293 producer cell supernatant can be either used directly or purified by ultrafiltration and/or ultracentrifugation.
(TIF)

S2 Fig Lack of IgG-binding capacity of CD16b(F) and IL-2::2Ig(F)GPI due to mutation of tryptophan W112. (**A**) HEK-293 producer cells were transfected with constructs as indicated. 72 hours later cells were analyzed for IL-2 (left column) and CD16b (middle column) surface expression. Figure displays staining with specific antibody (solid black line) or control antibody (dashed line). In addition, 5x10^5^ transfectants were incubated with pooled IgG (beriglobin P) followed by staining with an anti-human Ig-specific antiserum (right column, black solid line). Anti-human Ig-specific antiserum alone served as negative control (right column, dashed line). Numbers indicate percent positive cells. (**B**) Immunoblot analysis of VNP preparations obtained from HEK-293 cells transfected with MoMLV *gagpol* as particle inducing devices and IL-2v constructs as indicated. Anti-p30Gag was used as VNP loading control. Data are representative of four (A) and two (B) independent experiments.
(TIF)

S3 Fig Strong effector functions induced by IL-2::2Ig(F)GPI asVNP in the absence of bystander activation and proliferation. P14 splenocytes were labeled with CFSE proliferation dye and stimulated with 8 μg IL-2v asVNP as indicated. (**A and B**) After 96 hours cells were incubated with PMA/ionomycin in the presence of GolgiStop for 6 hours. Cells were subsequently stained with CD8- and TCR Vα2-specific mAb followed by intracellular IFN-γ staining and subjected to flow cytometric analysis. (**A**) Diagram depicts the fraction of IFN-γ-producing CD8^+^ TCR Vα2^+^-cells obtained from splenocyte cultures. (**B**) Density plots display intracellular IFN-γ-expression of CD8^-^ TCR Vα2^-^ lymphocytes relative to cellular proliferation as detected by CFSE-dilution. Markers were set according to negative control staining and non-proliferating cells. (**C**) Histogram overlays display surface expression of CD107a (LAMP1) (black solid line) or control mAb (shaded grey histogram) of CD8^+^ TCR Vα2^+^ cells after 72 hours of IL-2v asVNP co-culture. Untreated cells and cells stimulated with optimal amounts of LCMV-GP_33-41_ peptide (100 ng/ml) served as controls. Data are representative (B, C) or show the summary (A) of five (A, B), and one (C) experiments. * p < 0.05. ANOVA and Tukey’s multiple comparison test (A).
(TIF)

S4 Fig Antigen-specific down-regulation of CD127 expression upon co-incubation of CD8^+^ T cells with IL-2v asVNP. (**A**) Re-expression kinetics of CD127 on IL-2v asVNP stimulated purified P14 CD8^+^ TCR Vα2^+^ T cells. (**A**) Flow cytometry analysis of CD127 expression (black solid line) on purified P14 CD8^+^ T cells were stimulated with IL-2::GPI or IL-2::2Ig(F)GPI asVNP and analyzed at indicated time points for surface expression of CD127. Overlay histograms show staining with CD127-specific (black solid line) and Grey shaded histograms show staining with control antibodies. Numbers indicate mean fluorescence intensity. (**B**) Purified P14 CD8^+^ T cells were co-incubated with IL-2(A)::GPI and IL-2(A)::2Ig(F)GPI asVNP or IL-2::GPI and IL-2::2Ig(F)GPI ansVNP for three days and analyzed for surface expression of the high-affinity IL-7R, CD127. Overlay histograms show staining with CD127-specific (black solid line) and control antibody (grey shaded histogram). Numbers indicate mean fluorescence intensity. (**C**) Flow cytometry analysis showing expression of the indicated markers on naïve (shaded grey histograms) and *in vitro* pre-activated (solid black histograms) CD8^+^CD45.2^+^ donor cells isolated from the spleens of recipient mice. Data are representative (A-C) of three (except two for ansVNP in B) experiments or of 18 mice (eight per group, except for naïve (two), IL-2::GPI (three), αCD3/αCD28 plus IL-2 (five)) analyzed in two independent experiments.
(TIF)

S1 Table List of primers. The underlined regions indicate the restriction enzyme (RE) sites
(DOCX)

S2 Table List of mAbs. Abbreviations: APC, allophycocyanin; BV, brilliant violet; FITC, fluorescein isothiocyanate; Cy, cyanine; PE, phycoerythrin; HRP, horseradish peroxidase;
(DOCX)
