# Supplementary figures and images for: CD8+ T Cell Fate and Function Influenced by Antigen-Specific Virus-Like Nanoparticles Co-Expressing Membrane Tethered IL-2
Source: PLoS One. 2015 May 6;10(5):e0126034. doi: 10.1371/journal.pone.0126034 (PMC4422701; doi:10.1371/journal.pone.0126034)

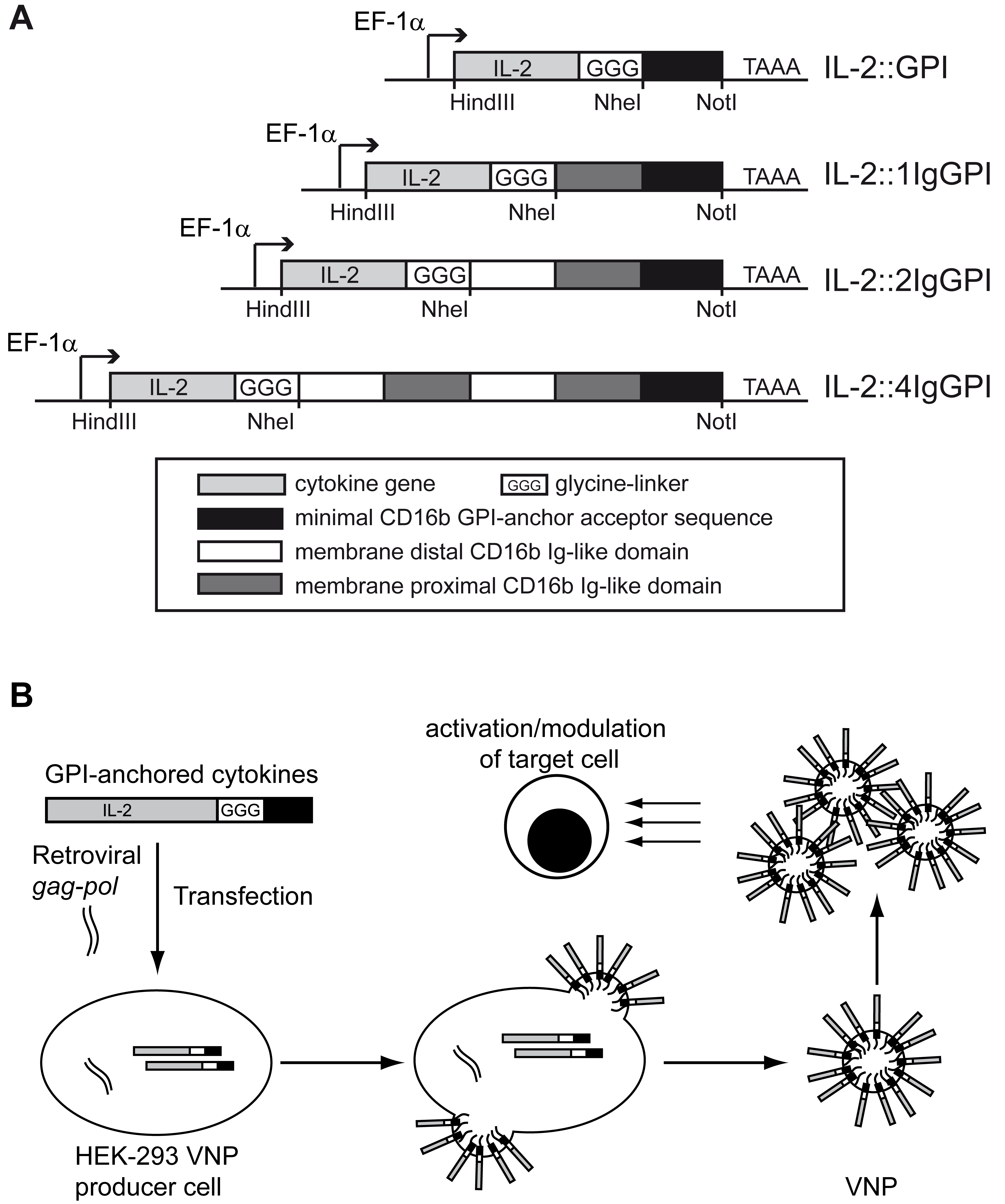

Supplement: S1 Fig — (A) Strategies for anchoring IL-2 to the plasma membrane. (B) Cytokines genetically fused to membrane anchors containing GPI-anchor acceptor sequences, are—upon expression in HEK-293 producer cells—targeted to lipid rafts of the plasma membrane. Formation of plasma membrane derived VNP is induced by co-transfection of producer cells with MoMLVgag-pol (OGP). Lipid raft resident molecules are incorporated into the VNP arising. In order to activate or modulate target cells, VNP-containing HEK-293 producer cell supernatant can be either used directly or purified by ultrafiltration and/or ultracentrifugation. (TIF) [file pone.0126034.s001.tif]

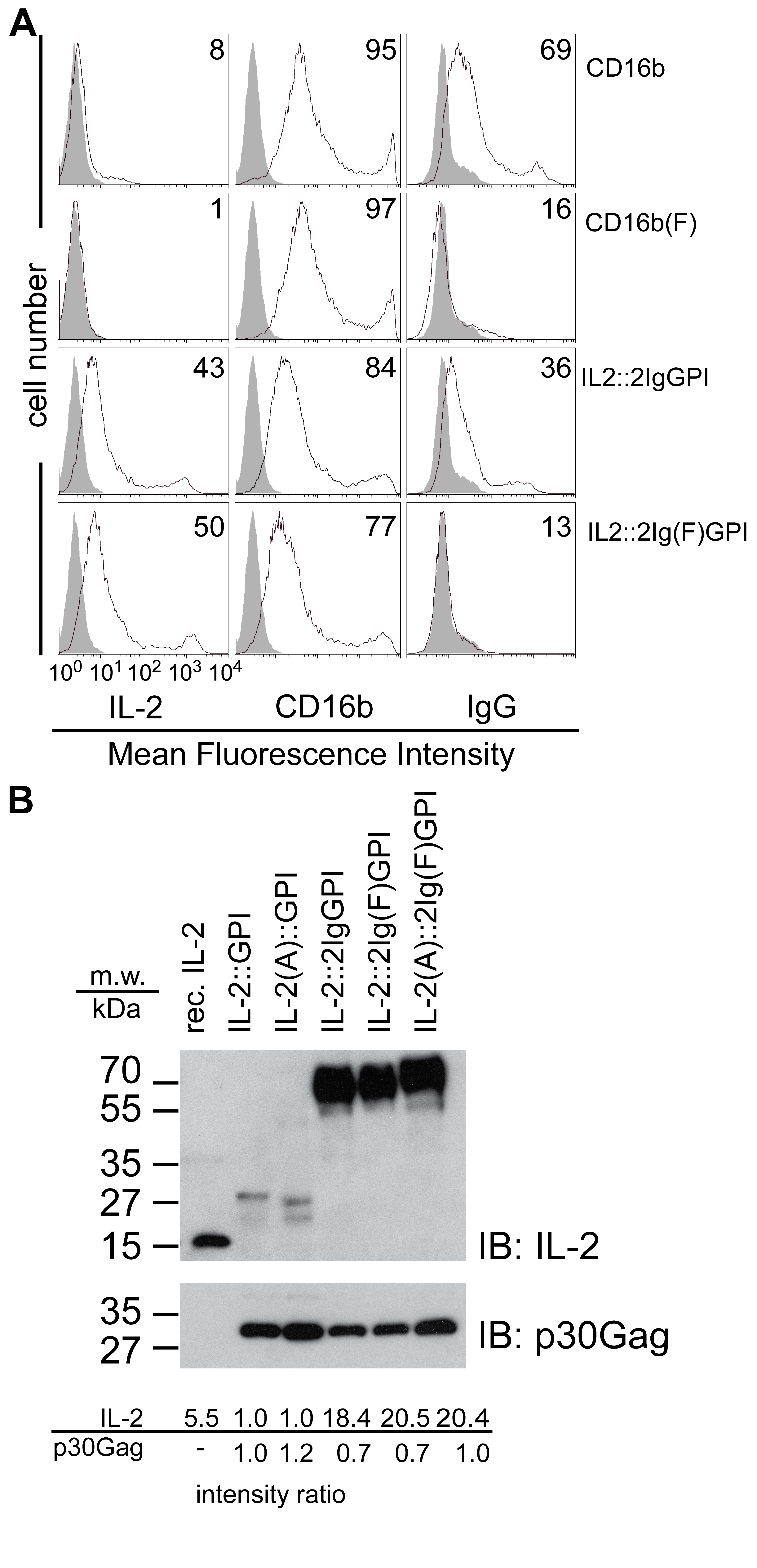

Supplement: S2 Fig — (A) HEK-293 producer cells were transfected with constructs as indicated. 72 hours later cells were analyzed for IL-2 (left column) and CD16b (middle column) surface expression. Figure displays staining with specific antibody (solid black line) or control antibody (dashed line). In addition, 5x10^5^ transfectants were incubated with pooled IgG (beriglobin P) followed by staining with an anti-human Ig-specific antiserum (right column, black solid line). Anti-human Ig-specific antiserum alone served as negative control (right column, dashed line). Numbers indicate percent positive cells. (B) Immunoblot analysis of VNP preparations obtained from HEK-293 cells transfected with MoMLV gagpol as particle inducing devices and IL-2v constructs as indicated. Anti-p30Gag was used as VNP loading control. Data are representative of four (A) and two (B) independent experiments. (TIF) [file pone.0126034.s002.tif]

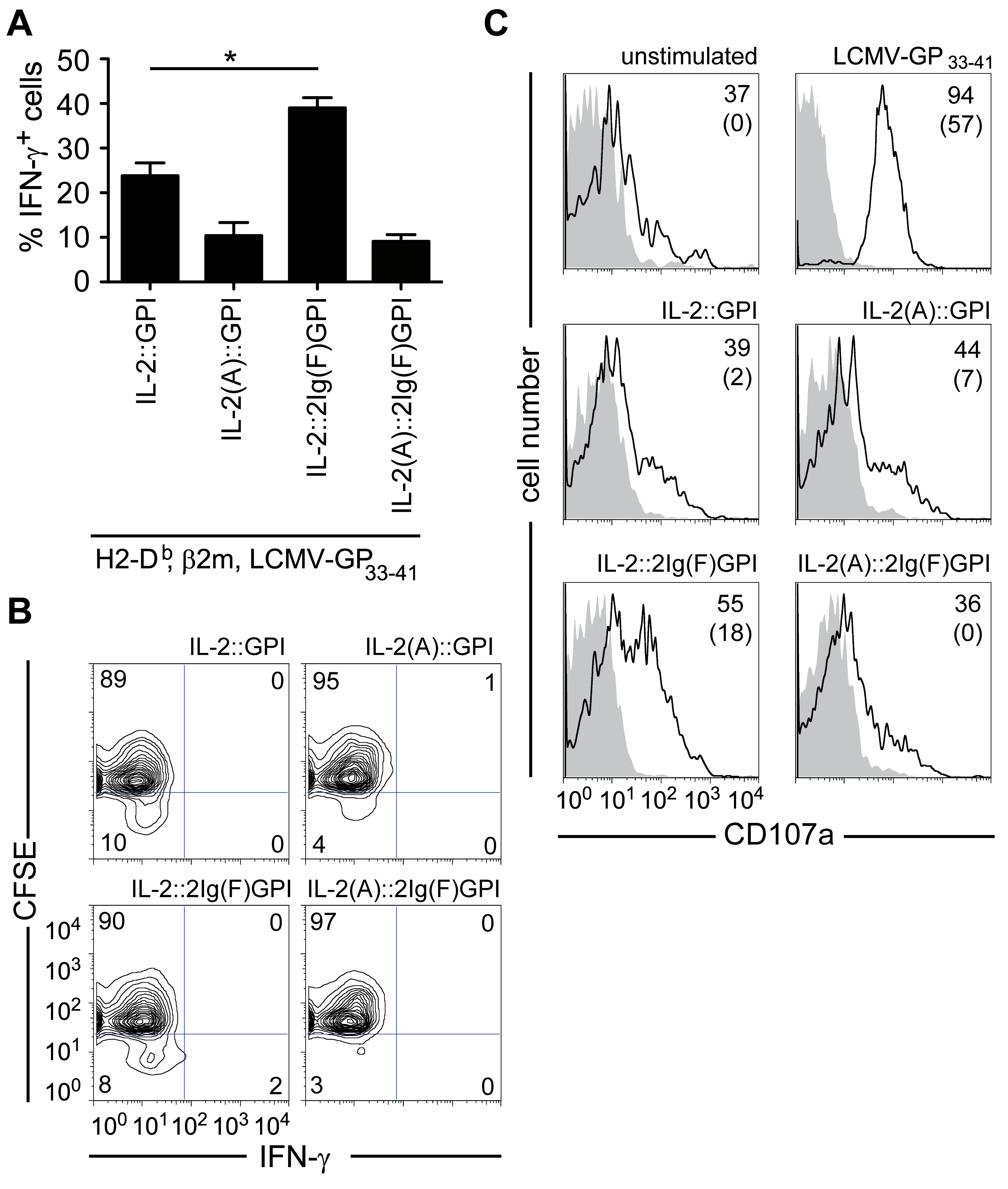

Supplement: S3 Fig — P14 splenocytes were labeled with CFSE proliferation dye and stimulated with 8 μg IL-2v asVNP as indicated. (A and B) After 96 hours cells were incubated with PMA/ionomycin in the presence of GolgiStop for 6 hours. Cells were subsequently stained with CD8- and TCR Vα2-specific mAb followed by intracellular IFN-γ staining and subjected to flow cytometric analysis. (A) Diagram depicts the fraction of IFN-γ-producing CD8+ TCR Vα2+-cells obtained from splenocyte cultures. (B) Density plots display intracellular IFN-γ-expression of CD8- TCR Vα2- lymphocytes relative to cellular proliferation as detected by CFSE-dilution. Markers were set according to negative control staining and non-proliferating cells. (C) Histogram overlays display surface expression of CD107a (LAMP1) (black solid line) or control mAb (shaded grey histogram) of CD8+ TCR Vα2+ cells after 72 hours of IL-2v asVNP co-culture. Untreated cells and cells stimulated with optimal amounts of LCMV-GP33-41 peptide (100 ng/ml) served as controls. Data are representative (B, C) or show the summary (A) of five (A, B), and one (C) experiments. * p < 0.05. ANOVA and Tukey’s multiple comparison test (A). (TIF) [file pone.0126034.s003.tif]

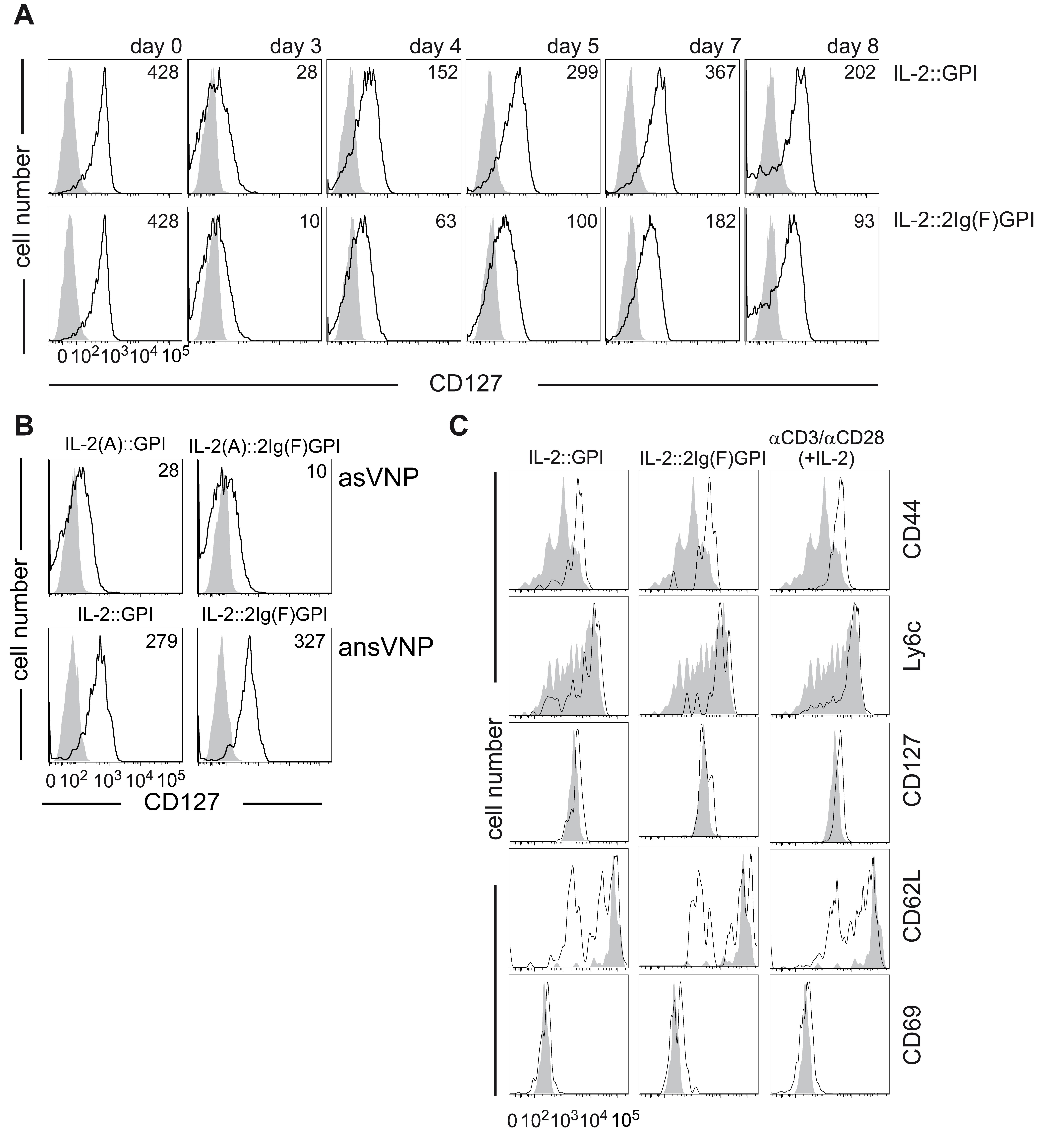

Supplement: S4 Fig — (A) Re-expression kinetics of CD127 on IL-2v asVNP stimulated purified P14 CD8+ TCR Vα2+ T cells. (A) Flow cytometry analysis of CD127 expression (black solid line) on purified P14 CD8+ T cells were stimulated with IL-2::GPI or IL-2::2Ig(F)GPI asVNP and analyzed at indicated time points for surface expression of CD127. Overlay histograms show staining with CD127-specific (black solid line) and Grey shaded histograms show staining with control antibodies. Numbers indicate mean fluorescence intensity. (B) Purified P14 CD8+ T cells were co-incubated with IL-2(A)::GPI and IL-2(A)::2Ig(F)GPI asVNP or IL-2::GPI and IL-2::2Ig(F)GPI ansVNP for three days and analyzed for surface expression of the high-affinity IL-7R, CD127. Overlay histograms show staining with CD127-specific (black solid line) and control antibody (grey shaded histogram). Numbers indicate mean fluorescence intensity. (C) Flow cytometry analysis showing expression of the indicated markers on naïve (shaded grey histograms) and in vitro pre-activated (solid black histograms) CD8+CD45.2+ donor cells isolated from the spleens of recipient mice. Data are representative (A-C) of three (except two for ansVNP in B) experiments or of 18 mice (eight per group, except for naïve (two), IL-2::GPI (three), αCD3/αCD28 plus IL-2 (five)) analyzed in two independent experiments. (TIF) [file pone.0126034.s004.tif]
