# Supplementary material for: CD8+ T Cell Fate and Function Influenced by Antigen-Specific Virus-Like Nanoparticles Co-Expressing Membrane Tethered IL-2
Source: PLoS One. 2015 May 6;10(5):e0126034. doi: 10.1371/journal.pone.0126034 (PMC4422701; doi:10.1371/journal.pone.0126034)
Supplement: S1 Table — The underlined regions indicate the restriction enzyme (RE) sites (DOCX) [file pone.0126034.s005.docx]

**S1 Table. List of primers**

| **Primer** | **Sequence ^a^** | **RE site** |
| --- | --- | --- |
| IL-2 for | 5′- CCCGCGAAGCTTGCCACCATGTACAGCATGCAGCTCGC-3' | *Hind* III |
| IL-2 rev | 5′- CGCGGGGCTAGCGCCGCCGCCTTGAGGGCTTGTTGAGATGATG -3' | *Nhe* I |
| 1IgGPI for | 5'- CGCGGGGCTAGCctctccaccctcagtgacc -3' | *Nhe* I |
| 2IgGPI for | 5'- CGCGGGGCTAGCcggactgaagatctcccaaag -3' | *Nhe* I |
| CD16b rev | 5'- CGCGGGGCGGCCGCTTTAAATGTTTGTCTTCACAGAGAAATATAGT -3' | *Not* I |
| 4IgGPI rev | 5'- CGCGGGACTAGTACCTTGAGTGATGGTGATGTTC -3' | *Spe* I |
| IL-2mut for | 5'- GATCTTCAGGCCCTAGAAGATG -3' |  |
| IL-2mut rev | 5'- CATCTTCTAGGGCCTGAAGATC -3' |  |
| 16bmut for | 5'- GTGTCACAGCTTTAAGAACACTGCTCTGC-3' |  |
| 16bmut rev | 5'- CTCAGGTGAATAGGTTCTTCCTCCTTGAAC -3' |  |
| pEAK12 for | 5'- CATTCTCAAGCCTCAGACAGTGG -3' |  |
| pEAK12 rev | 5'- TGGATGCAGGCTACTCTAGGG -3' |  |
