# Supplementary material for: CD8+ T Cell Fate and Function Influenced by Antigen-Specific Virus-Like Nanoparticles Co-Expressing Membrane Tethered IL-2
Source: PLoS One. 2015 May 6;10(5):e0126034. doi: 10.1371/journal.pone.0126034 (PMC4422701; doi:10.1371/journal.pone.0126034)
Supplement: S2 Table — Abbreviations: APC, allophycocyanin; BV, brilliant violet; FITC, fluorescein isothiocyanate; Cy, cyanine; PE, phycoerythrin; HRP, horseradish peroxidase; (DOCX) [file pone.0126034.s006.docx]

**S2 Table. List of mAbs**

| **Specificity** | **Clone name** | **Species** | **Conjugation** | **Source** |
| --- | --- | --- | --- | --- |
| **Flow cytometry:** |  |  |  |  |
| CCR7 | 4B12 | rat | BV421 | Biolegend, San Diego, CA, USA |
| CD8a | 5H10 | rat | APC | Caltag, Burlingham, CA, USA |
| CD16b | 3G8 | mouse | PE | Molecular Probes, Eugene, OR, USA |
| CD25 | PC61 | rat | FITC | Biolegend, San Diego, CA, USA |
| CD44 | IM7 | rat | BV510 | BD Pharmingen™, San Jose, CA, USA |
| CD45.1 | A20 | mouse | PE | eBioscience, San Diego, CA, USA |
| CD62L | MEL-14 | rat | APC | Biolegend, San Diego, CA, USA |
| CD69 | H1.2F3 | hamster | FITC | Caltag, Burlingham, CA, USA |
| CD127 | A7R34 | rat | eFluor® 450 | eBioscience, San Diego, CA, USA |
| IFN-γ | XMG1.2 | rat | APC | eBioscience, San Diego, CA, USA |
| IL-2 | JES6-5H4 | rat | PE | Caltag, Burlingham, CA, USA |
| Ly6c | AL-21 | rat | FITC | BD Pharmingen™, San Jose, CA, USA |
| TCR Vα2 | B20.1 | rat | PE | BD Pharmingen™, San Jose, CA, USA |
| control | MOPC-21 | mouse | APC | BioLegend, San Diego, CA, USA |
| control | 4H1-A7/VIAP | mouse | PE/FITC | An der Grub, Kaumberg, Austria |
|  |  |  |  |  |
| **Immunoblotting** |  |  |  |  |
| IL-2 | polyclonal | rabbit |  | PeproTech, London, UK |
| CD59 | MEM-43/5 | mouse |  | Exbio, Praha, Cech Republic |
| CD147 | MEM-M6/6 | mouse |  | Exbio, Praha, Cech Republic |
| MoMLV p30 Gag | R187 | rat |  | ATCC, Manassas, VA, USA |
| rabbit Ig | polyclonal | goat | HRP | DAKO, Glostrup, Denmark |
| mouse Ig | polyclonal | goat | HRP | DAKO, Glostrup, Denmark |
| rat Ig | polyclonal | rabbit | HRP | DAKO, Glostrup, Denmark |
